# Impact of Environmental Conditions on YOLOv8-Based Traffic Sign Detection: A Controlled Comparison of Simulated and Real Weather Cases

**DOI:** 10.3390/s26185843

**Published:** 2026-09-15

**Authors:** Ziyad N. Aldoski, Csaba Koren, Daniel Miletics

**Affiliations:** 1 Department of Highway and Bridge, Technical College of Engineering, Duhok Polytechnic University, 61 Zakho Road, Duhok 1006, Kurdistan Region, Iraq; 2 Department of Transport Infrastructure and Water Resources Engineering, Széchenyi István University, Egyetem tér 1, 9026 Győr, Hungary; mileticsd@sze.hu

**Keywords:** autonomous vehicle, traffic sign detection, weather simulation, YOLOv8, representative detection confidence, environmental robustness, road safety

## Abstract

Robust traffic sign detection is essential for reliable autonomous driving systems; however, detection performance can be substantially affected by adverse environmental conditions. Although simulation-based approaches are widely used to evaluate robustness, the extent to which simulated weather reproduces real-world environmental effects remains insufficiently understood. This study presents a controlled, comparative evaluation methodology for assessing a YOLOv8-based traffic sign detection model across four environmental conditions: clear (sunny), simulated rain, real-world rain, and simulated snow. The same road segment, camera configuration, and predefined set of 55 traffic-sign instances were maintained across the evaluated conditions, enabling interpretable comparisons while minimizing scene-level variability. Detection performance was assessed using representative detection confidence (RDC) at the traffic-sign-instance level, along with image-quality metrics such as sharpness, saturation, and intensity. Mean RDC was highest under clear conditions (0.825), followed descriptively by simulated snow (0.647), real-world rain (0.408), and simulated rain (0.395). However, simulated and real-world rain did not differ significantly in RDC (Holm-adjusted *p* = 0.770), while the Friedman test indicated a significant overall difference among conditions (χ^2^(3) = 98.767, *p* < 0.001; Kendall’s W = 0.599). Image-quality analysis further revealed substantial differences between rainfall conditions in successfully detected traffic-sign regions, particularly in Sharpness and Mean Saturation. Overall, the findings demonstrate that simulated weather can produce traffic-sign-level detector responses that are statistically comparable to those observed under independently recorded real-world rainfall, while producing substantially different image-level characteristics. The results support the use of controlled weather simulation as a complementary evaluation approach, alongside real-world validation, to investigate the environmental robustness of camera-based traffic-sign detection systems.

## 1. Introduction

Traffic sign detection constitutes a fundamental component of perception systems in autonomous vehicles (AVs), enabling the interpretation and enforcement of regulatory, warning, and guidance information in real-world driving environments [1]. Accurate recognition of traffic signs is essential for safe navigation, as these elements directly influence vehicle behavior, including speed control, maneuvering, and hazard response.

In recent years, vision-based detection systems leveraging deep learning have achieved significant success in traffic sign recognition tasks, thanks to their ability to learn complex visual representations from large-scale data [2]. Among these approaches, the YOLO (You Only Look Once) family of algorithms has emerged as a prominent solution, offering an effective balance between detection accuracy and real-time computational efficiency [2,3]. The latest iteration, YOLOv8, introduces architectural improvements that enhance real-time object detection performance and confidence estimation, making it particularly suitable for real-time applications in autonomous driving systems [4,5].

Despite these advancements, the robustness of deep learning-based traffic sign detection models under adverse environmental conditions remains a critical challenge. Weather-related factors such as rain, snow, fog, and reduced visibility introduce complex visual distortions, including Gaussian blur, occlusion, contrast attenuation, and illumination variability, all of which degrade image quality and hinder reliable detection [6,7]. These impairments can significantly compromise the performance of vision-based perception systems, leading to missed detections or incorrect classifications and posing safety risks in real-world autonomous driving scenarios [8].

Several studies have investigated traffic sign detection under adverse environmental conditions by proposing improved detection architectures, lightweight networks, or data augmentation strategies [9,10,11,12,13]. Although these studies have demonstrated considerable progress, their evaluation methodologies often rely on unconstrained real-world datasets or on independently generated synthetic data, in which environmental conditions change simultaneously with scene composition, camera viewpoint, traffic density, and other contextual factors. Consequently, it becomes difficult to determine whether observed performance variations are due to environmental degradation itself or to unrelated scene-level variability. From a transportation engineering perspective, this limitation restricts the ability to establish experimentally grounded relationships between environmental conditions and perception reliability in safety-critical driving environments [14,15].

Our previous studies investigated traffic sign detection under illumination variability [16] and general weather-related environmental conditions [17]. These studies demonstrated the influence of environmental factors on detection performance. Still, they did not provide a controlled experimental framework that could directly compare simulated and real-world weather conditions while preserving identical scene characteristics. As a result, the realism of simulation-based environmental transformations and their ability to reproduce real-world degradation effects remained difficult to assess.

To address this research gap, this study proposes a controlled-comparative evaluation methodology to assess the robustness of YOLOv8-based traffic sign detection under adverse environmental conditions. The proposed controlled-comparative evaluation methodology systematically varies environmental degradation while preserving identical road geometry, traffic sign instances, camera configuration, temporal structure, and the predefined S1–S55 traffic-sign inventory across all evaluated scenarios. By minimizing scene-level variability, the proposed controlled-comparative evaluation methodology enables an interpretable assessment of weather-associated changes in detector response while providing an experimentally reproducible comparison between simulated and real-world environmental conditions.

The proposed controlled-comparative evaluation methodology is implemented using the ZND dataset [16], whose construction and validation are described in detail in the original publication. Building upon this dataset, the proposed controlled-comparative evaluation methodology incorporates a custom weather-simulation framework that generates simulated rain and snow conditions from clear-weather video sequences by applying Gaussian blur, occlusion, contrast reduction, saturation loss, and intensity variation. This controlled experimental design enables experimentally reproducible evaluation of environmental robustness while maintaining identical scene characteristics across all evaluated conditions.

Beyond quantitative performance evaluation, the proposed controlled-comparative evaluation methodology also enables investigation of the mechanisms through which environmental degradation affects detection reliability. The controlled experimental design facilitates interpretation of how visual degradations such as blur, contrast attenuation, saturation reduction, and localized occlusions influence detection confidence performance by reducing edge sharpness, texture consistency, and feature visibility. These degradations are likely to degrade convolution-based feature extraction and localization confidence, particularly for small or partially occluded traffic signs.

The principal contribution of this work is the development and validation of a controlled-comparative evaluation methodology for experimentally assessing the robustness of YOLOv8-based traffic sign detection under adverse environmental conditions. By systematically varying environmental degradation while preserving identical scene geometry, traffic sign instances, camera configuration, temporal structure, and the predefined S1–S55 traffic-sign inventory, the proposed methodology enables a controlled and interpretable analysis of changes in detector response associated with environmental degradation. It provides a systematic and experimentally reproducible comparison between simulated and real-world environmental conditions. Using this methodological framework, the study further investigates how different weather conditions affect the confidence of representative detection, image quality, distributional variability across traffic-sign instances, and perceptual reliability, providing interpretable insights into the mechanisms by which environmental degradation affects vision-based autonomous driving systems.

### Main Contributions

The primary contribution of this study is the development of a controlled-comparative evaluation methodology to assess the robustness of traffic sign detection under adverse environmental conditions. Unlike conventional robustness evaluations that rely on unconstrained datasets or isolated synthetic augmentation, the proposed framework systematically varies environmental degradation while preserving identical scene geometry, traffic sign instances, camera configuration, temporal structure, and the predefined S1–S55 traffic-sign inventory. This design enables a controlled assessment of environmental effects on detector response and an experimentally reproducible comparison between simulated and real-world environmental conditions.

Using this methodological framework, the study further provides the following supporting contributions:

- Development of a custom weather simulation pipeline capable of generating controlled simulated rain and snow conditions through the integration of Gaussian blur, occlusion, contrast reduction, saturation loss, and intensity variation.
- Comparative evaluation between simulated and real-world rain conditions to assess the realism and limitations of synthetic environmental transformations.
- Integration of representative detection confidence (RDC) with image-quality indicators (sharpness, saturation, and intensity) to establish interpretable relationships between visual degradation and detector response.
- Comprehensive robustness analysis integrating nonparametric statistical validation using the Friedman test and Wilcoxon signed-rank post hoc tests with Holm correction, object-level detection evaluation, distributional analysis across traffic-sign instances, and image-quality assessment to explain the relationship between environmental degradation and representative detection confidence.

Rather than proposing a new detection architecture, this work contributes to transportation safety engineering by providing a rigorous experimental methodology for evaluating environmental robustness. The proposed framework provides an interpretable basis for understanding how weather-induced degradation affects perceptual reliability and supports the development of more reliable vision-based autonomous driving systems.

The remainder of this paper is organized as follows. Section 2 reviews related work on traffic sign detection and environmental robustness. Section 3 presents the methodology, including dataset description, weather simulation techniques, and evaluation metrics. Section 4 reports and discusses the experimental results, including descriptive and inferential statistical analyses, distributional analysis of representative detection confidence, image-quality assessment, and object-level robustness evaluation. Section 5 presents the conclusions, followed by the study limitations and future research directions.

## 2. Literature Review

### 2.1. Importance of Traffic Sign Detection for Autonomous Vehicles

Traffic sign detection plays a vital role in the perception systems of autonomous vehicles (AVs), requiring both high accuracy and real-time performance under diverse environmental conditions. Recent advances in deep learning, particularly the YOLO (You Only Look Once) family of object detection models, have demonstrated strong performance in controlled environments [16]. However, environmental challenges such as rain, fog, and snow continue to undermine detection reliability, often compromising the operational safety of AVs. These conditions introduce noise, occlusion, and visual distortion, challenging even state-of-the-art vision-based models and highlighting the need for more robust, resilient detection approaches.

In our previous work, detection performance was evaluated under varying illumination conditions, specifically comparing daytime and nighttime scenarios [16]. That study demonstrated the significant influence of lighting variability on detection accuracy. However, it did not address the impact of weather-induced environmental degradation, which remains a critical factor in the real-world deployment of autonomous driving systems.

Traffic-sign detection is also directly relevant to transportation engineering because reliable interpretation of regulatory, warning, and guidance signs is associated with road-user safety, vehicle operation, and the effectiveness of intelligent transportation systems. Related research in the industrial Internet of Things and mobile edge computing has also examined intelligent resource allocation mechanisms for connected computational environments, highlighting the broader importance of efficient, reliable intelligent infrastructure in emerging transportation and IoT systems [18]. From this perspective, environmental robustness should not be considered solely as a computer-vision performance problem; it is also an important transportation-safety consideration because weather-induced perception degradation can affect the reliability of information available to automated driving systems.

Accordingly, recent research has increasingly examined traffic-sign detection under challenging environmental conditions, including adverse weather, illumination variability, small-object characteristics, and partial occlusion [5,6,9,13,14]. These studies demonstrate that detection performance depends not only on the selected detection architecture but also on the interaction between environmental conditions, sign characteristics, image quality, and scene context.

### 2.2. Performance of YOLO Models Under Adverse Conditions

YOLO-based models have undergone numerous adaptations to improve detection-confidence performance under challenging environmental conditions [19,20]. Previous research has shown that weather variations significantly affect model performance [21]. For example, Seraj et al. [9] reported a substantial reduction in detection success under snowy and cloudy conditions, with a 56% drop in performance. Although recall rates remained relatively high in cloudy (84.11%) and snowy (81.93%) conditions, detecting low-contrast or partially occluded signs remained problematic.

Similarly, Feng and Jia [6] demonstrated that environmental distortions such as Gaussian blur significantly reduce detection accuracy, particularly for underrepresented traffic sign categories. Their findings highlight the combined impact of dataset imbalance and insufficient modeling of environmental factors, leading to increased false negatives and reduced reliability in safety-critical applications.

Building on these findings, our recent work further investigated the impact of adverse environmental conditions on traffic sign detection performance [17]. While that study examined the general effects of environmental degradation, it did not provide a controlled comparison between simulated and real-world weather conditions, a key focus of the present research.

More recent studies have continued to demonstrate the sensitivity of traffic-sign detectors to adverse environmental conditions. For example, Li et al. [22] proposed DSF-YOLO for robust multiscale traffic-sign detection in adverse weather conditions and reported improved detection performance through architectural and feature-fusion modifications. Similarly, Li et al. [23] developed an efficient YOLOv11-based approach incorporating scale-aware feature processing and scene-perception mechanisms for traffic-sign detection under adverse weather conditions. These studies demonstrate the ongoing efforts to improve detector architecture and training strategies for challenging environments.

However, these architecture-oriented studies primarily seek to improve detector performance by modifying the detection model itself. In contrast, the present study addresses a different methodological question: how consistently does a fixed, previously trained detector respond when environmental conditions are systematically varied while scene-level characteristics are preserved? This distinction is important because improved detector architecture and robust evaluation methodology represent complementary but different research objectives.

### 2.3. Algorithmic Innovations for Robust Detection

To address these limitations, several studies have proposed architectural enhancements and model adaptations. For instance, TS-YOLO incorporates targeted data augmentation strategies, including fog and snow simulation, achieving a 33.11% improvement in mean average precision (mAP@0.5) [12].

Similarly, Sign-YOLO extends the YOLOv7 framework by integrating attention mechanisms and squeeze-and-excitation (SE) blocks, improving feature discrimination and achieving a mAP of 99.10% with reduced computational complexity [24].

Beyond YOLO-based approaches, two-stage object-detection architectures have also been investigated for traffic-sign detection, particularly for small and low-resolution signs. Cao et al. proposed an improved Faster R-CNN framework incorporating modified bounding-box regression, improved RoI processing, multiscale feature fusion, and an improved NMS strategy, demonstrating improved detection of small objects, including traffic signs [25].

Despite these advancements, challenges remain. Alshahrani et al. [14] reported that although YOLOv8 achieved high overall mAP (95.3%) under fog and rain conditions, performance variability persisted across sign types and visibility levels. Interestingly, traditional convolutional models such as VGG-16 outperformed YOLOv8 in classification accuracy (98.68%), suggesting that modern detection architectures remain susceptible to environmental degradation.

Further improvements have been proposed through lightweight architectures and attention-based feature fusion modules, which enhance the detection of small and occluded signs while maintaining computational efficiency [5]. Nevertheless, robust detection under adverse environmental conditions remains a persistent challenge across existing models [26].

Recent research has also explored transformer-based architectures to improve perceptual robustness in intelligent transportation systems. For example, Tian et al. proposed a Dynamic Transformer Network (DTNet) for vehicle detection, integrating dynamic convolution and mixed-attention mechanisms to enhance adaptability under challenging conditions, such as illumination variations and occlusions [27]. Although this work addresses vehicle rather than traffic-sign detection, it illustrates the broader trend toward adaptive perception architectures in intelligent transportation applications.

Similarly, recent work on vehicular edge computing has investigated dependency-aware service caching and task offloading to support intelligent connected-vehicle environments, illustrating the broader integration of computational intelligence within modern intelligent transportation systems [28].

Beyond computer vision applications, recent artificial intelligence research has combined particle swarm optimization with large language models to automate and improve task-specific information extraction, demonstrating the broader use of optimization-driven AI methodologies across application domains [29].

Taken together, the literature demonstrates a clear trend toward improving the detector itself through attention mechanisms, feature fusion, lightweight architecture, multiscale processing, and transformer-based components [5,12,22,23,24]. Nevertheless, improving the architecture does not by itself establish whether a detector has been evaluated under sufficiently controlled environmental conditions. Comparisons between synthetic and natural weather can remain confounded when changes in scene geometry, camera viewpoint, traffic composition, illumination, or sign instances accompany weather changes.

The present study, therefore, complements architecture-oriented research rather than competing directly with it. The YOLOv8s detector is kept fixed, and the experimental emphasis is placed on the controlled evaluation of environmental robustness. This allows the study to investigate environmental effects without simultaneously introducing changes to the detector architecture or training procedure.

### 2.4. Physical Properties of Traffic Signs and Visibility Factors

Beyond algorithmic considerations, the physical properties of traffic signs play a critical role in detection-confidence performance. Studies by Saleh and Fleyeh [15,30], along with recent perception-focused analyses [31], highlight the importance of retroreflectivity, color composition, and material properties in determining sign visibility under varying lighting and weather conditions.

Their findings demonstrate that factors such as sheeting type, color contrast, and sign category significantly influence detectability. However, accurately quantifying these effects remains challenging, as there are no universally accepted thresholds for defining visibility degradation or operational obsolescence.

This line of research underscores that detection-confidence performance depends not only on model architecture but also on the interaction between environmental degradation and the physical characteristics of traffic signs.

This interaction is particularly relevant for transportation engineering because traffic signs are physical roadside assets whose visibility depends on both their intrinsic characteristics and the surrounding environmental conditions. Factors such as retroreflective properties, color composition, sign size, orientation, illumination, and surface condition may influence the visual information available to camera-based perception systems [1,15,25,26].

Consequently, evaluating environmental robustness using only aggregate detector-level metrics may obscure differences among individual traffic-sign categories or visual characteristics. This motivates the object-level and color-composition analyses conducted in the present study, which examine whether a fixed detector’s response varies across different physical and visual sign characteristics.

### 2.5. Synthetic Data and Simulation for Adverse Weather Conditions

Due to the scarcity and high cost of collecting real-world adverse weather data, simulation and synthetic data generation have emerged as important tools for evaluating AV perception systems.

Recent studies have demonstrated the effectiveness of synthetic datasets and simulation platforms in augmenting training data and enabling controlled experimentation. For example, the SYNTHIA dataset [32] provides large-scale synthetic urban scenes with pixel-level annotations, improving detection robustness when combined with real-world data. Similarly, the CARLA simulator [33] supports customizable environmental conditions, enabling systematic evaluation across diverse scenarios.

In addition, prior research has demonstrated the broader applicability of artificial intelligence in transportation infrastructure analysis. For instance, Antwi et al. [34,35] employed AI-driven object detection methods to identify roadway features and school zones in aerial imagery, highlighting the potential of data-driven methods for large-scale transportation monitoring. However, these studies also highlight a persistent limitation: the lack of large-scale, well-annotated datasets that represent rare or hazardous environmental conditions.

Collecting such datasets under real-world adverse environmental conditions remains logistically challenging, resource-intensive, and often unsafe, further motivating the use of simulation-based approaches for controlled evaluation.

Despite these advantages, simulation fidelity remains a critical challenge. While physics-based rendering and generative approaches improve realism, discrepancies between simulated and real-world conditions persist. Recent transportation simulation studies have also emphasized that controlled experimental designs are essential for improving the interpretability of simulation-based evaluations by minimizing confounding environmental and scene-level factors [36]. Accordingly, simulation should not automatically be regarded as an equivalent substitute for natural environmental conditions; rather, its validity depends on the purpose of the experiment and on the extent to which the simulated transformation reproduces the characteristics relevant to the research question.

The SSIM can be used as an image-level descriptor to quantify structural changes between a reference image and a transformed image. However, SSIM primarily characterizes structural similarity between the images being compared and should not, by itself, be interpreted as a direct measure of physical weather realism or of equivalence to natural weather conditions. In the present study, SSIM is therefore used to characterize the magnitude of structural modification introduced by the simulated weather transformations rather than to establish physical equivalence between simulated and real-world weather.

This distinction is important for the controlled-comparative methodology adopted here. The objective is not to demonstrate that the simulated weather is physically identical to natural rainfall or snowfall, but rather to determine whether controlled environmental transformations can produce detector-level responses that are comparable to those observed under independently recorded real-world weather.

Thus, the comparison between simulated and real-world weather is treated as a validation-oriented comparison of detector response and image characteristics rather than as a claim of complete physical realism. This approach allows the simulation framework to be evaluated in terms of its usefulness for controlled robustness experiments while explicitly recognizing the limitations of synthetic weather generation.

In this context, the present study adopts a controlled-comparative evaluation methodology supported by a controlled weather simulation framework that introduces controlled visual degradations representative of selected adverse-weather characteristics while maintaining experimental reproducibility.

### 2.6. Research Gap and Contribution of This Study

Although researchers have conducted extensive studies on traffic sign detection, most prior work focuses primarily on conventional performance metrics such as precision, recall, and mean average precision.

These conventional metrics remain essential for evaluating detector accuracy when suitable manually annotated bounding-box ground truth is available. However, they do not, by themselves, explain how environmental degradation modifies the visual information available to a detector or how the detector’s response varies across predefined physical traffic sign instances.

A second limitation concerns experimental comparability. Many studies evaluate different weather conditions using independently acquired datasets or images. Under such designs, differences in road geometry, traffic composition, camera position, illumination, sign instances, and background context may occur simultaneously with changes in weather. Consequently, observed differences in detector performance cannot always be attributed specifically to environmental degradation.

Furthermore, although previous work—including our own—has explored the effects of illumination [16] and general weather variability [17], these studies did not provide a controlled and systematic comparison between simulated and real-world environmental conditions. This limitation restricts a deeper understanding of how well simulation-based evaluations reflect real-world performance.

A further gap is therefore methodological rather than architectural. There is a need for an evaluation framework that, as far as is practically possible, isolates environmental variation from scene-level variation and subsequently compares detector responses under controlled synthetic transformations with independently recorded natural weather conditions.

The present study addresses this gap through a controlled comparative evaluation methodology rather than by developing a new detection architecture. The previously trained YOLOv8s detector is kept fixed, while environmental conditions are varied across clear, simulated rain, real-world rain, and simulated snow. The same road segment, camera configuration, and predefined physical traffic-sign inventory are retained for the controlled comparisons involving the same reference sequence.

The principal supporting contributions of the study are:

- Evaluating detector response using RDC at the predefined traffic-sign-instance level, together with image-quality metrics including sharpness, saturation, and intensity.
- Investigating whether environmental degradation produces heterogeneous responses across individual traffic-sign categories and color-composition groups.
- Comparing simulated and independently recorded real-world rain conditions to examine whether synthetic weather transformations produce detector-level responses that are broadly comparable to natural-weather observations, while explicitly recognizing the limitations of the independent recording sessions.
- Providing a controlled experimental design in which environmental transformations can be evaluated while preserving the same road geometry, camera configuration, temporal structure, and predefined traffic-sign inventory for the controlled weather conditions.
- Integrating nonparametric statistical analysis, object-level evaluation, distributional analysis, and image-quality assessment to provide complementary evidence regarding environmental robustness.

Unlike studies that propose new detection modules and validate them through architecture-level ablation experiments, the present work does not introduce a new neural-network architecture or competing detector modules. Therefore, its methodological contribution lies in the experimental evaluation framework rather than in architectural modification of YOLOv8.

The proposed framework should consequently be viewed as complementary to existing SOTA detector-development studies [5,12,21,22,23]. While those studies primarily seek to improve detection accuracy through architectural or training innovations, the present study investigates how a fixed detector behaves when environmental conditions are systematically varied.

This distinction also defines the scope of the present work. The study does not claim superiority over alternative detection architectures, nor does it seek to establish a universally optimal traffic-sign detector. Instead, it provides an experimentally controlled methodology for examining environmental robustness and for identifying differences between simulated and real-world weather effects.

By integrating computer vision evaluation with transportation engineering perspectives, this work provides an experimentally reproducible framework for investigating environmental robustness in autonomous driving perception systems.

## 3. Methodology

This section presents the experimental framework used to evaluate the performance of the YOLOv8 traffic-sign detection model under varying environmental conditions. The methodology is designed to ensure reproducibility, analytical rigor, and controlled comparison across scenarios. It includes the study design, dataset, environmental conditions, detection model, evaluation metrics, and statistical analysis procedures.

A key feature of the study is the use of a controlled experimental setup in which the same road segment, camera configuration, and set of physical traffic-sign instances are maintained across the evaluated environmental conditions. This design reduces scene-level variability and enables a more direct and interpretable assessment of the effects of weather-induced visual degradation on detection performance.

### 3.1. Study Design

This study employs a controlled-comparative methodology to evaluate the robustness of a YOLOv8-based traffic-sign detection model under different environmental conditions. The design minimizes scene-level variability by using the same predefined road segment, physical traffic-sign instances, camera configuration, and direction of travel across the evaluated scenarios.

The clear-weather recording serves as the reference sequence for generating simulated rain and simulated snow, thereby preserving the underlying scene content and temporal structure. An independently recorded natural rain sequence is included as the real-world rain condition. Thus, four environmental conditions are evaluated: clear (sunny), simulated rain, simulated snow, and real-world rain.

A previously trained YOLOv8s model, developed using the ZND dataset [16], is applied without retraining, fine-tuning, or architectural modification. The evaluation focuses on changes in representative detection confidence (RDC) and associated image-quality characteristics across the four conditions. Frame-level detections are consolidated into predefined traffic-sign instances before statistical analysis, with the highest-confidence detection retained as the representative.

By maintaining consistent traffic-sign instances and inference procedures across conditions, the framework enables a more direct and interpretable comparison of simulated and naturally occurring environmental degradation while reducing the influence of scene-level differences. The simulated conditions provide greater experimental control, whereas the independently recorded real-world rain conditions provide a natural-weather comparison subject to recording-session variability.

### 3.2. Data Collection

To evaluate traffic-sign detection under varying environmental conditions, video data were collected using a controlled acquisition procedure. The recordings were captured with a high-resolution Stereolabs ZED2i stereo camera (Stereolabs, Paris, France) mounted on a Lexus RX450h (Toyota Motor Corporation/Lexus, Cambridge, ON, Canada) along a predefined road segment in urban and semi-urban areas of Győr, Hungary.

Two video conditions were acquired: clear daytime recordings (clear condition) and natural-rain recordings (real-world rain condition), using the same vehicle platform and camera configuration. The real-world rain recording was conducted on 19 February 2026, whereas the clear-weather reference recording was conducted on 6 March 2026, resulting in a 15-day interval between the two recording sessions. The availability of suitable natural rainfall determined this interval, as the real-world rain recording was intentionally conducted under actual rainy conditions and therefore required waiting for an appropriate rainfall event. The clear-weather recording was conducted at approximately 10:15 a.m. under natural daylight and clear, bright illumination. In contrast, the real-world rain recording was conducted at approximately 10:30 a.m. under natural daylight and rainy, overcast conditions. Illumination was assessed qualitatively from the recorded scenes because no calibrated illuminance measurements were collected. Traffic density and precipitation intensity were likewise not quantitatively measured during the recording sessions.

The clear-weather recording was repeatedly inspected to identify traffic signs that were visibly presented to the camera and suitable for evaluation. Signs facing the opposite direction or substantially obscured by vegetation or other scene elements were excluded. This procedure identified 55 observable physical traffic-sign instances, labeled S1–S55 in the order of their appearance along the road segment.

These 55 physical signs formed the predefined evaluation inventory and were not manually annotated as frame-level ground-truth bounding boxes. Their physical presence was established independently of detector output through visual inspection of the reference recording. Before model processing, the first author coded the S1–S55 inventory by sign type, physical location, and sequential order in the reference video. Following YOLOv8s inference, detected signs were manually associated with the predefined inventory using their predicted class, visual appearance, spatial location, sequential position, and video timestamp. The complete association was then rechecked against the processed video and detection output at least twice for each environmental-condition video, including both detected and non-detected predefined signs. Because the predefined inventory and video sequence provided known sign identities and locations, the association could be verified consistently across the four evaluation conditions.

For each sign and environmental condition, multiple frame-level detections of the same physical sign were consolidated into a single representative observation by selecting the highest-confidence valid detection. The resulting confidence value was used as the RDC, while image-quality measurements were retained from the same frame.

The clear-weather recordings were also used to generate the simulated rain and simulated snow sequences described in Section 3.4. Because these sequences originated from the same reference recordings, their road geometry, traffic-sign instances, camera viewpoint, driving direction, and temporal scene structure were preserved. The simulated sequences, therefore, retained the underlying illumination and scene characteristics of the clear-weather recordings, while the applied weather transformations modified their visual appearance. In contrast, the real-world rain recordings were independently acquired on the same road segment using the same vehicle and camera configuration. Although they shared the same S1–S55 inventory, they inevitably included recording-session variability in factors such as illumination, traffic conditions, and scene appearance, thereby providing less experimental control than the simulated conditions.

This acquisition and evaluation procedure provided a consistent basis for comparing detector behavior under clear conditions, simulated rain, simulated snow, and real-world rain, while minimizing scene-level variability within the controlled simulated-weather comparisons.

### 3.3. ZND Dataset for Training

The YOLOv8s detection model used in this study was previously trained on the publicly available ZND dataset developed in our prior work [16]. Because the dataset construction, annotation procedure, model training, and baseline evaluation have been comprehensively documented in [16], only the information relevant to the present evaluation is summarized here. No new training, retraining, fine-tuning, or model adaptation was performed in this study; the previously trained YOLOv8s model was applied directly to the evaluation videos described in Section 3.2.

**Figure 1:**
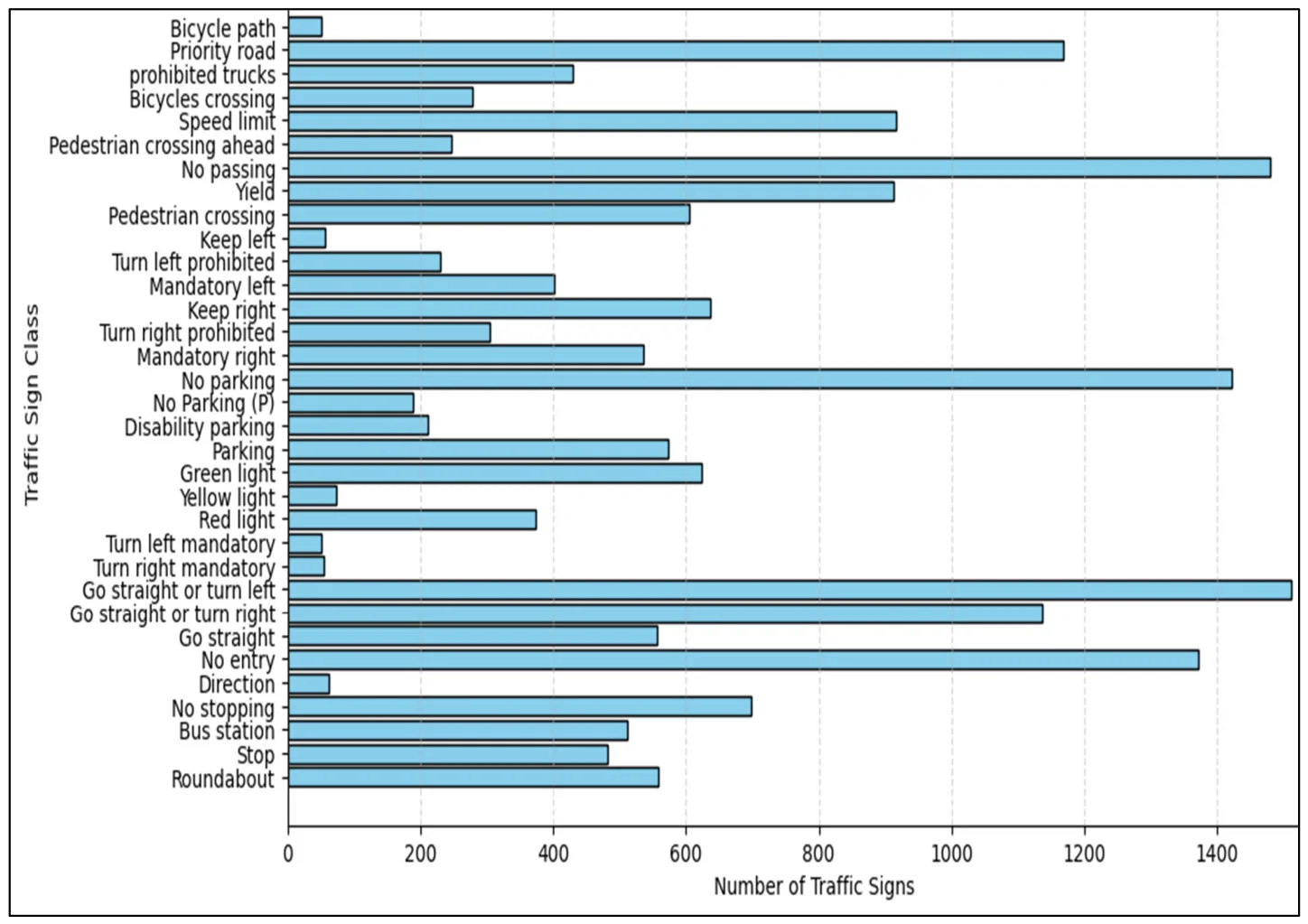
Distribution of traffic sign classes in the ZND dataset used for YOLOv8 model training and validation. The figure illustrates the number of annotated samples available for each traffic sign category.

The ZND dataset contains approximately 16,500 annotated traffic-sign images across 33 classes, including regulatory, warning, and guidance signs. It combines images from the German Traffic Sign Recognition Benchmark (GTSRB), publicly available image repositories, and real-world traffic-sign images collected and annotated by the authors. Images were manually annotated using LabelImg v1.8.6 and converted to the YOLO object-detection format. Dataset quality was enhanced through manual annotation verification, retention of clearly identifiable signs, and class-balancing strategies. Figure 1 presents the distribution of traffic-sign samples across the dataset.

The ZND dataset is publicly available via [https://www.kaggle.com/datasets/endaziar/znd-dataset (accessed on 20 April 2026)], enabling reproducibility and comparative evaluation by other researchers. Additional information regarding dataset construction, annotation methodology, quality control procedures, and YOLOv8 training configuration is available in [16].

To avoid data leakage, the evaluation route used in the present study was separate from the locations represented in the author-collected component of ZND, and no images of the 55 physical traffic-sign instances (S1–S55) evaluated in this study were included in the training dataset. The presence of the same traffic-sign categories, or visually similar signs, in ZND does not constitute reuse of the evaluation instances. The 55 predefined physical signs were therefore reserved exclusively for the present robustness evaluation. The simulated rain and snow sequences were generated from the clear-weather evaluation recording and were likewise used only for evaluation.

### 3.4. Weather Simulation Techniques

To evaluate the robustness of traffic-sign detection, a modular weather-simulation framework was developed to generate controlled synthetic rain and snow sequences from the clear-weather videos described in Section 3.2. The framework preserves the original road geometry, traffic-sign instances, camera configuration, and frame sequence while introducing controlled visual degradations representing selected perceptual characteristics of adverse weather.

The framework was implemented in Python 3.14.5 using OpenCV 4.13.0.92 and NumPy 2.4.6. Environmental degradation was introduced through global and localized transformations, including Gaussian blur, partial occlusion, contrast reduction, color desaturation, and intensity variation. The final simulation parameters, including fixed values and randomized ranges where applicable, are summarized in Table 1. These parameters were predefined and applied consistently to the corresponding video sequences to ensure reproducibility while maintaining the underlying scene content and traffic-sign locations.

Unlike conventional image-augmentation methods primarily intended to increase training diversity, the proposed framework was designed exclusively for evaluation and was not used for training, retraining, or fine-tuning the detector. The generated sequences were used to assess detector robustness under controlled environmental degradation.

**Figure 2:**
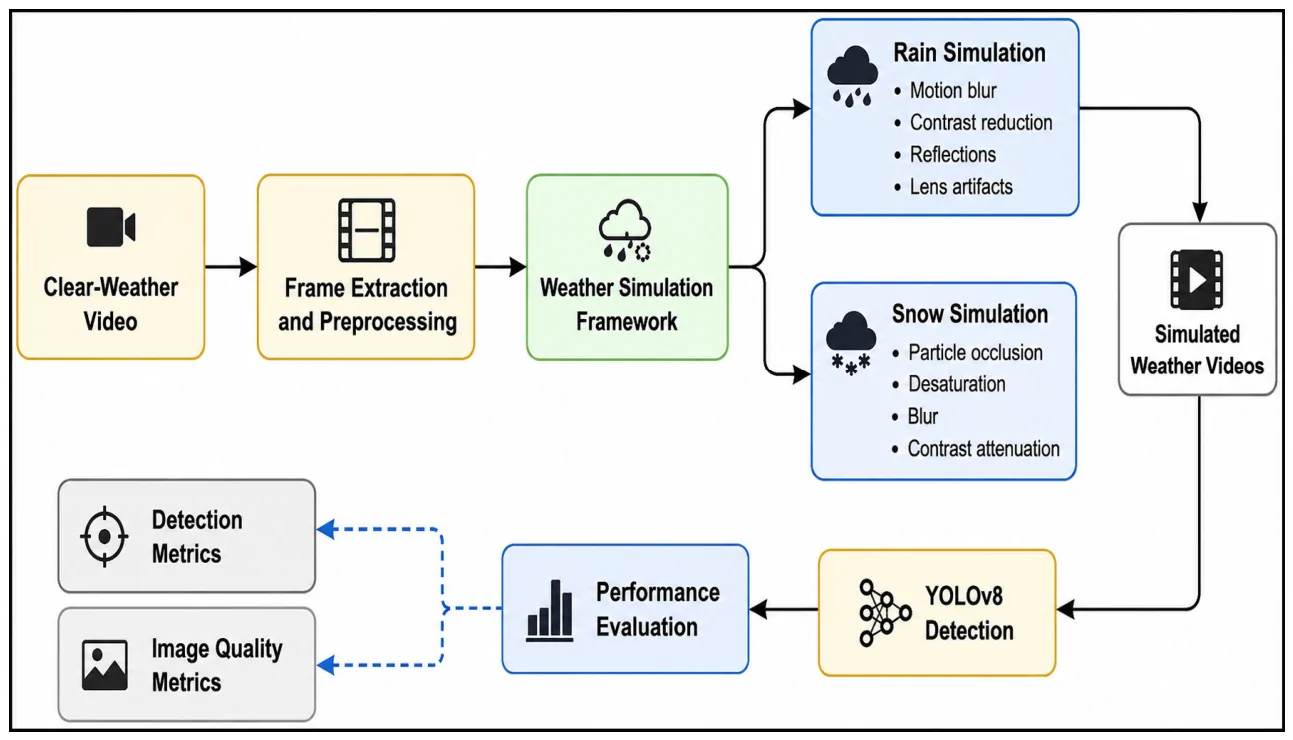
Overview of the proposed weather simulation and evaluation framework.

Figure 2 presents the proposed weather simulation and evaluation framework. The pipeline comprises frame extraction, preprocessing, weather simulation, synthetic video generation, YOLOv8s traffic-sign detection, and performance evaluation using detection and image-quality metrics. The framework includes two simulation modules: simulated rain and simulated snow.

#### 3.4.1. Rain Simulation

The rain simulation module generates a controlled representation of moderate rainfall by superimposing semi-transparent, slanted rain streaks onto each video frame. The position, orientation, length, and thickness of the rain streaks were randomized to introduce spatial variability while preserving the underlying scene content and traffic-sign locations of the clear-weather reference sequence.

To simulate the principal visual effects associated with rainfall, the simulation incorporates several sequential image transformations, including rain-streak generation, localized lens-water effects, visibility attenuation, a localized wet-road effect, global image blurring, and small image displacement. Each transformation was applied to a defined image layer or processing stage to distinguish and reproduce the role of the corresponding parameter.

First, the rain-streak layer was generated by introducing approximately 1000 semi-transparent slanted streaks per frame. The streak length was randomly varied between 10 and 20 pixels, with a thickness of 2 pixels and a slant randomly selected from −10 to −4 pixels. A 5 × 5 Gaussian blur was applied to the rain-streak layer to soften the individual streaks and represent their appearance under rainfall and camera motion.

Second, localized lens-water effects were introduced to represent water droplets on the camera lens. Thirty-five randomly positioned droplets were generated per frame, with droplet radii ranging from 10 to 30 pixels and opacity values sampled between 0.10 and 0.30. The resulting lens-water layer was Gaussian-blurred with a 9 × 9 kernel (σ = 5) before being blended with the frame. This blur operation was applied specifically to the lens-water layer and is therefore distinct from the 5 × 5 blur applied to the rain-streak layer and the subsequent global blur used to degrade visibility.

Third, visibility degradation was introduced using a fog-based transformation with an intensity of 0.40 to represent the diffuse reduction in visibility associated with rainfall. This transformation was applied to the processed frame after the rain-streak and lens-water layers had been introduced.

Fourth, a localized wet-road effect was generated using 15 randomly positioned line elements in the lower region of the image to represent wet pavement and surface reflections. The elements were blended with a weight of 0.10 and incorporated into the processed frame before the final global image-degradation stage.

Finally, global image degradation was introduced via Gaussian blurring with an 11 × 11 kernel (σ = 3), followed by a small, randomized image displacement of ±2 pixels in the horizontal and vertical directions. This global blur was applied to the processed image to represent broader image softening associated with reduced visibility and water-related image degradation.

Accordingly, the overall processing sequence consisted of the clear-weather input frame, generation and blending of the rain-streak layer, generation and blending of the lens-water layer, application of fog-based visibility degradation, addition of the localized wet-road effect, global Gaussian blurring, and final image displacement. The resulting frame was then used as the synthetic-rain evaluation frame. These transformations were intended to reproduce selected visual consequences of rainfall, including reduced visibility, image softening, and wet-road appearance, rather than to constitute a physically complete model of atmospheric rainfall [37].

#### 3.4.2. Snow Simulation

The snow simulation module generates a controlled representation of snowfall by introducing randomly distributed particles representing airborne snowflakes together with diffuse visibility degradation. The snow particles were varied in size, opacity, and apparent motion to reproduce selected visual characteristics of snowfall while preserving the underlying scene content and traffic-sign locations of the clear-weather reference sequence.

Because snowfall typically produces more diffuse visual degradation than the directional streak patterns associated with rainfall, the simulation emphasizes soft particle occlusion, mild Gaussian blur, reduced contrast, and color desaturation.

The synthetic snow sequences were generated using 700 snow particles per frame, with particle radii randomly varied between 2 and 6 pixels, consistent with the final simulation parameters reported in Table 1. A 5 × 5 Gaussian blur was applied to the snow-particle layer. Image displacement of ±2 pixels in the horizontal and vertical directions was introduced to represent small variations in image position associated with environmental and camera motion. The additional implementation parameters, including lens-frost characteristics, fog intensity, global Gaussian blur settings, and image displacement range, are summarized in Table 1.

The simulation additionally incorporated 30 randomly positioned lens-frost specks per frame, with radii ranging from 8 to 25 pixels and opacity values sampled from 0.10 to 0.25. The resulting lens-frost layer was Gaussian-blurred using an 11 × 11 kernel with σ = 3. A winter-fog intensity of 0.40 was applied, followed by global Gaussian blurring using a 9 × 9 kernel with σ = 2.5. The global blur was applied to the processed frame to represent diffuse visibility degradation under snowy conditions. These transformations were applied to reproduce selected visual characteristics of snowfall and winter-visibility degradation while preserving the underlying scene structure.

Although the proposed simulation reproduces several visual characteristics of snowfall, including airborne particle occlusion and reduced visibility, it does not model snow accumulation on traffic signs, partial obstruction of sign boundaries, or long-term environmental deposition. Consequently, the simulated snow sequences represent controlled visual approximations of snowfall-related degradation rather than complete physical models of natural snowfall.

#### 3.4.3. Simulation Output and Experimental Conditions

The proposed framework generated two synthetic evaluation datasets from the clear-weather reference recordings: simulated rain and simulated snow. Together with the original clear-weather recordings and the independently acquired real-world rain recordings, these datasets constitute the four environmental conditions evaluated in this study:

- Clear (sunny)
- Simulated rain
- Real-world rain
- Simulated snow

The simulated rain and simulated snow sequences were generated directly from the clear-weather reference recordings. Consequently, the underlying road geometry, traffic-sign instances, camera configuration, driving direction, and temporal sequence were preserved in simulated-condition comparisons. This design allows changes in detector response to be examined under controlled visual degradation while minimizing changes in the underlying scene content.

In contrast, the real-world rain sequence was independently recorded on the same predefined road segment, using the same vehicle platform and camera configuration, and included the same predefined traffic-sign inventory. However, because it was acquired during a separate recording session, the real-world rain condition cannot be considered frame-level equivalent to the clear-weather reference. Differences in traffic context, illumination, vehicle motion, and other uncontrolled factors may therefore be present.

Accordingly, the simulated conditions provide the highest degree of experimental control. In contrast, real-world rain conditions provide a natural-weather reference against which detector behavior under actual rainfall can be examined. The four conditions are therefore used jointly to characterize detector response under controlled and naturally occurring environmental degradation, while the results involving real-world rain are interpreted with appropriate caution.

Within the simulated-condition comparisons, environmental degradation represents the intentionally varied experimental factor, while the underlying scene content and temporal sequence remain unchanged. This controlled structure provides the principal basis for examining the effect of simulated environmental degradation on traffic-sign detection response while minimizing changes in the underlying scene content. Comparisons involving real-world rain are interpreted as comparisons between independently acquired experimental conditions rather than as estimates of the isolated causal effect of rainfall.

#### 3.4.4. Validation and Limitations of Simulation

The structural effects of the simulated weather transformations were evaluated using the SSIM between the clear-weather reference images and their corresponding simulated images. Because the simulated rain and simulated snow sequences were generated directly from the clear-weather recordings, SSIM could be calculated on corresponding frames while preserving the same underlying scene content, traffic-sign instances, camera configuration, and temporal structure.

SSIM was used to quantify the degree of image-level structural modification introduced by the simulated weather transformations. The resulting SSIM values are reported in Section 4. Importantly, SSIM was used to characterize structural similarity between corresponding reference and simulated images and was not interpreted as a measure of physical weather realism or perceptual equivalence to natural weather.

The proposed framework intentionally represents controlled environmental degradation rather than a complete physical simulation of adverse weather. Real-world rainfall introduces additional effects, including lens contamination, localized reflections, spatially varying illumination, water accumulation, and other dynamic optical effects that cannot be fully reproduced through image-based transformations. Likewise, the snow simulation does not account for snow accumulation on traffic-sign surfaces, persistent snow deposition, or structural occlusion caused by accumulated snow.

Accordingly, the independently recorded real-world rainfall sequence was incorporated as a natural-weather reference condition rather than as a ground-truth validation set for the simulated rain. This allows the study to examine detector behavior under both controlled simulated degradation and naturally occurring rainfall while maintaining a clear distinction between the two experimental settings.

The comparative analysis presented in Section 4, therefore, evaluates differences in detector response and image characteristics across the four environmental conditions rather than claiming that the simulated rain or snow reproduces the physical processes of natural weather. The simulation should consequently be understood as a controlled experimental representation of selected visual effects of adverse weather, rather than as a physically complete weather model.

### 3.5. Detection Model and Experimental Setup

The object detection model employed in this study is YOLOv8s, selected in the previous work [16] for its favorable balance between computational efficiency and detection accuracy, as well as its suitability for real-time traffic-sign detection applications. In the present study, YOLOv8s was used as a fixed, previously trained detector to evaluate the effects of environmental degradation, rather than to develop, retrain, fine-tune, or otherwise modify the detection model.

The YOLOv8s model used in this study was previously trained using the ZND dataset described in Section 3.3 and introduced in [16]. No additional training, retraining, fine-tuning, or model adaptation was performed in the present study. The previously trained model was loaded and applied directly to the evaluation videos under all four environmental conditions. The original model development and training procedures are documented in [16].

The evaluation was conducted under four environmental conditions: clear, simulated rain, real-world rain, and simulated snow. The same fixed YOLOv8s model, model weights, and inference procedure were applied consistently across all four conditions. Inference was performed using the prediction defaults of the Ultralytics 8.3.36 implementation, including a confidence threshold of 0.25, an NMS IoU threshold of 0.70, and an input image size of 640 pixels; no study-specific inference overrides were specified. The inference device was selected automatically by the Ultralytics framework. The simulated rain and simulated snow sequences were used exclusively for evaluation and were not introduced into the model during its original training or subsequently during the present study.

For the simulated conditions, the underlying road geometry, traffic-sign instances, camera configuration, driving direction, and temporal sequence were preserved from the corresponding clear-weather recordings. Consequently, comparisons among the clear, simulated rain, and simulated snow conditions provide the highest level of experimental control for examining the detector’s response to simulated environmental degradation. The real-world rain condition was independently recorded on the same road segment using the same vehicle platform and camera configuration, and it contained the same predefined traffic-sign inventory. Because this recording was acquired independently, complete frame-level equivalence with the clear-weather reference sequence cannot be assumed.

Accordingly, the experimental setup distinguishes between controlled simulated-weather comparisons and the independently recorded real-world rain condition. The simulated conditions provide controlled visual modifications of the same underlying scene, whereas the real-world rain condition provides a natural-weather reference for examining detector behavior under actual rainfall. This distinction is maintained throughout the subsequent statistical analysis and interpretation of the results.

### 3.6. Frame-Level Detection Processing and Final Evaluation Dataset

Each evaluation video was processed frame-by-frame using OpenCV, and the fixed pre-trained YOLOv8s model was applied through the Ultralytics YOLO implementation. For each processed frame, the detector generated predicted bounding boxes, class labels, and confidence scores for the traffic signs it recognized. The YOLOv8s inference was performed using the Ultralytics implementation’s default settings, with a confidence threshold of 0.25, an NMS IoU threshold of 0.70, and an input image size of 640 pixels; no study-specific overrides were specified. The resulting detection records were retained together with the corresponding frame number and frame time. The clear-weather, simulated-rain, and simulated-snow recordings each contained 10,620 frames over 354 s at 30 frames/s, whereas the real-world rain recording contained 10,200 frames over 340 s at 30 frames/s.

The evaluation was based on a predefined inventory of 55 traffic-sign instances identified through repeated visual inspection of the clear-weather recording. These physical traffic-sign instances were assigned unique identifiers (S1–S55) in the order of their appearance along the recorded road segment. No manual bounding-box annotations were created for these 55 signs. Instead, the identifiers were used to establish a consistent reference for the same physical traffic-sign instances across the environmental conditions.

After the S1–S55 evaluation inventory had been established, all evaluation videos were processed using the fixed YOLOv8s detector. The detection records were then examined alongside the processed video output to determine whether each predefined traffic-sign instance (S1–S55) was detected under each environmental condition. Because the evaluation inventory represented physical traffic-sign instances rather than manually annotated frame-level bounding boxes, the correspondence between individual YOLOv8 detections and the predefined S1–S55 instances was established through manual examination of the processed video and detection output. Detections were grouped with the corresponding physical sign using the predicted traffic-sign class, spatial position in the scene, visual appearance, and sequential occurrence along the recorded road segment.

This manual association procedure was applied to distinguish repeated detections of the same physical traffic sign across consecutive frames from detections corresponding to different traffic-sign instances. The selected associations were reviewed against the processed video to ensure that the detection corresponded to the intended physical traffic-sign instance. If the physical traffic sign was present in the scene but the model produced no corresponding detection during its period of visibility, it was classified as an undetected instance for that environmental condition.

Because the evaluation videos contain continuous sequences, the same physical traffic sign may be detected repeatedly over multiple consecutive frames. Treating all such detections as independent observations would give greater weight to signs that remain visible for longer periods, potentially inflating the effective sample size. Therefore, a representative-observation selection procedure was applied to obtain a single observation for each predefined traffic-sign instance under each environmental condition.

For each detected traffic-sign instance, all frame-level detections manually associated with that physical sign were compared according to their YOLOv8s detection confidence scores. The observation with the highest YOLOv8s confidence score was selected as the representative observation, and its confidence value was recorded as the RDC. The selected observation was retained, along with all image-quality and environmental metrics calculated for the same frame and the detected sign region. Thus, the detection response and the corresponding visual characteristics always originated from the same observation. If a predefined traffic-sign instance was not detected in any frame under a particular environmental condition, its RDC was recorded as zero in the final evaluation dataset to represent detector failure for a traffic-sign instance known to be present. Image metrics that require a detected bounding box were not artificially assigned to these non-detected cases.

The same processing and selection procedure was applied consistently to all four environmental conditions. Consequently, the final evaluation dataset represents the detection responses of the fixed YOLOv8s detector for the same predefined S1–S55 traffic-sign inventory under each condition, while avoiding repeated counting of the same traffic sign across consecutive frames.

#### 3.6.1. Representative Observation Selection

The representative observation was selected separately for each of the 55 predefined traffic-sign instances (S1–S55) under each environmental condition. After the evaluation videos were processed with YOLOv8s, the frame-level detections manually associated with each predefined physical traffic-sign instance were collected and compared using their confidence scores.

When a traffic sign was detected across multiple frames, the detection confidence values for all corresponding observations were compared, and the frame with the highest YOLOv8s confidence score was selected as the representative observation. The confidence score from this selected observation was then recorded as the RDC for the corresponding traffic-sign instance and environmental condition.

For example, if one traffic-sign instance was detected in 10 consecutive frames, with confidence values ranging from 0.61 to 0.95, the highest-confidence observation (0.95) was selected as the representative. All other metrics associated with that same frame, including Overall Intensity, Legend Intensity, Background Intensity, Sharpness, Mean Saturation, and Occlusion Ratio, were retained. The metrics were therefore not selected independently from different frames.

If a predefined traffic-sign instance was not detected in any frame under a particular environmental condition, its RDC was recorded as zero. This zero value indicates that the YOLOv8s detector failed to produce a corresponding detection for a predefined traffic-sign instance known to be present in the evaluation scene. No image-quality or bounding-box-based measurements were assigned to the non-detected instance because no corresponding detector bounding box was available.

This procedure resulted in at most one representative observation for each traffic-sign instance under each environmental condition. The selected confidence value was used as the RDC for that sign-condition combination. Importantly, RDC represents the maximum YOLOv8s detection confidence observed during the available observation of a predefined traffic-sign instance. It is therefore a traffic-sign-level representative confidence measure and should not be interpreted as a conventional frame-level detection rate, detection probability, recall, or precision measure.

The resulting dataset was subsequently used for comparative and statistical analyses.

#### 3.6.2. Final Evaluation Dataset

Following the representative-observation selection procedure, the detection records from the four environmental conditions were consolidated into a final evaluation dataset. The dataset was organized around the 55 predefined physical traffic-sign instances (S1–S55) rather than around individual video frames.

For each environmental condition, each detected traffic-sign instance contributed one representative observation corresponding to the frame with the highest detection confidence. The associated image quality and environmental measurements were taken from that same observation. For a traffic-sign instance known to be present in the scene but not detected in any frame, the RDC was recorded as 0, and metrics that require a detected sign region were treated as unavailable.

Accordingly, the final evaluation dataset was structured to provide one observation for each of the 55 predefined traffic-sign instances across the four environmental conditions (clear, simulated rain, real-world rain, and simulated snow), yielding a maximum of 220 sign-condition observations. A complete observation was therefore defined by the traffic-sign identifier (S1–S55), environmental condition, selected representative frame, RDC, and the image-quality measurements available for that observation.

Thus, the final dataset provides a consistent traffic-sign-level representation of detector performance across the four environmental conditions. This approach prevents multiple consecutive detections of the same physical traffic sign from being treated as independent observations. It ensures that each predefined traffic-sign instance contributes at most one comparable observation per environmental condition to the environmental-condition comparison.

All subsequent statistical analyses, including the Friedman test and Wilcoxon signed-rank post hoc comparisons, were performed using this final traffic-sign-level dataset rather than the complete frame-level detection records. The frame-level records were used only to identify detections, associate them with the predefined physical traffic-sign instances, and select the representative observation.

### 3.7. Evaluation Metrics

The evaluation framework combined the detector’s representative detection response with complementary image-quality and environmental measurements extracted from the same selected observation. These measurements were used to characterize both changes in traffic-sign detection performance and the visual conditions associated with each environmental scenario.

Because the study evaluates predefined traffic-sign instances observed in continuous video sequences, the final analysis was conducted at the traffic-sign-instance level after the representative-observation selection procedure described in Section 3.6. Specifically, the 55 predefined target traffic-sign instances, coded S1–S55, constituted the reference inventory for the analysis. This ensured that the same physical traffic-sign instances were evaluated across all four environmental conditions. This approach avoids treating repeated detections of the same physical traffic sign across consecutive frames as independent observations.

#### 3.7.1. Representative Detection Confidence

The primary detection performance measure was the highest YOLOv8s confidence score for each predefined traffic-sign instance under each environmental condition. For consistency with the terminology used throughout the analysis, tables, and figures, this variable is defined as the RDC. Accordingly, RDC represents a confidence-based measure of the detector’s response to a predefined traffic-sign instance and should not be interpreted as a conventional detection rate, recall, or precision metric.

During inference, a traffic sign may be detected in multiple consecutive frames, with the detector producing different confidence values across these observations. Rather than averaging these repeated detections, the detection with the highest confidence score was selected as the representative observation for the corresponding traffic-sign instance. The confidence score associated with this observation was recorded as the RDC.

For example, if a traffic sign was detected across multiple consecutive frames with confidence values of 0.61, 0.74, 0.83, and 0.95, the highest confidence value (0.95) was retained as the RDC for that traffic-sign instance. All image-quality metrics associated with the frame in which the 0.95 confidence score was obtained were retained from the same observation. This procedure ensured that the RDC and its associated image-quality measurements corresponded to the same traffic-sign instance and the same video frame.

When a predefined traffic-sign instance was present in the evaluation scene but not detected in any frame under a particular environmental condition, its RDC was set to zero. This zero value indicates that the YOLOv8s detector did not produce a corresponding valid detection meeting the specified confidence threshold of 0.25 for a predefined traffic-sign instance known to be present in the evaluation scene; it does not indicate the absence of the physical traffic sign. Image-quality measurements requiring a valid detection bounding box were not assigned to these cases.

The resulting RDC therefore provides a traffic-sign-level measure of the maximum YOLOv8s detection confidence observed during the visibility period of each predefined traffic-sign instance under each environmental condition. RDC is consequently a representative confidence measure rather than a conventional detection metric. This distinction is important because the present study uses RDC to characterize the strength of the detector’s response to predefined traffic-sign instances. In contrast, detector detection failure is represented separately by RDC = 0.

#### 3.7.2. Image and Environmental Quality Metrics

Several image-based metrics were extracted from the detected traffic-sign region corresponding to the representative observation. These measurements were taken from the same frame used to determine the highest detection response, thereby maintaining a direct correspondence between the RDC and the visual characteristics of the detected traffic sign.

Overall Intensity represents the mean pixel intensity within the detected traffic-sign region and indicates the overall brightness of the observed sign.

Legend Intensity represents the mean intensity of pixels classified as belonging to the brighter region within the detected traffic-sign region using the predefined intensity-thresholding procedure.

Background Intensity represents the mean intensity of the remaining pixels within the detected traffic-sign region after separation of the brighter region.

Sharpness was quantified using the Laplacian variance. This metric indicates local image detail and edge definition, with lower values generally corresponding to reduced edge sharpness and increased image blur.

Mean Saturation was calculated in the HSV color space and represents the average saturation of pixels within the detected traffic-sign region. Changes in saturation indicate variations in color characteristics associated with environmental conditions and illumination.

Occlusion Ratio represents the proportion of pixels within the detected traffic-sign region that fall below the predefined intensity threshold. It was used as an indicator of the proportion of the detected sign region characterized by low-intensity or potentially visually obscured areas. Importantly, these image-quality metrics were not independently selected to maximize or minimize their respective values. For each detected traffic-sign instance, all image-quality and environmental metrics were retained from the same representative observation selected according to the highest detection confidence. Consequently, the RDC value and its associated visual measurements correspond to the same traffic-sign instance and the same video frame.

#### 3.7.3. Structural Similarity Index Measure (SSIM)

The structural similarity index measure (SSIM) was used to quantify the visual and structural changes introduced by the simulated-weather transformations relative to the corresponding clear-weather reference frames. Because the simulated rain and simulated snow sequences were generated directly from the corresponding clear-weather recordings, frame-to-frame SSIM comparisons could be performed while preserving the same underlying scene content, traffic-sign instances, camera configuration, and temporal structure.

In this study, SSIM was used as an image similarity measure to quantify the extent of visual modification introduced by the simulated weather conditions. Higher SSIM values indicate greater structural similarity to the reference frame, whereas lower values indicate greater structural and visual modification. SSIM was therefore interpreted as a measure of visual alteration rather than physical weather realism or detection performance.

The independently recorded real-world rain condition was not included in the frame-to-frame SSIM comparison because it was acquired during a separate recording session and did not share the same frame-level temporal sequence or corresponding reference frames as the clear-weather recording. Accordingly, a direct SSIM comparison would not provide an equivalent controlled assessment.

The resulting SSIM values for the simulated rain and simulated snow conditions are reported in Section 4. Importantly, SSIM was not used to establish physical equivalence between the simulated and naturally occurring weather conditions, but rather to characterize the degree of image modification produced by the controlled simulation process.

### 3.8. Statistical Analysis

Statistical analysis was performed in Python using SciPy 1.17.1 on the final traffic-sign-level evaluation dataset described in Section 3.6 to compare the RDC and associated image-quality metrics across the four environmental conditions: clear, simulated rain, real-world rain, and simulated snow. Descriptive statistics, including mean, standard deviation, median, minimum, and maximum, were calculated for RDC and the available image-quality metrics. Box plots and violin plots were used to examine distributions, dispersion, and potential extreme observations.

Because the same predefined traffic-sign instances (S1–S55) were evaluated under all four conditions, RDC measurements were treated as related repeated observations. By reducing consecutive frame-level detections of each predefined traffic-sign instance to a single representative observation before inferential analysis, the analysis avoided treating temporally adjacent detections of the same physical sign as independent observations. Given the bounded nature of RDC (0–1), the presence of zero-valued observations indicating detector failures, and the non-normality of the paired observations, a nonparametric repeated-measures approach was adopted. The Friedman test was therefore used to assess overall differences among the four environmental conditions. When a significant omnibus difference was identified, pairwise Wilcoxon signed-rank tests were performed with Holm correction for the six pairwise comparisons. Statistical significance was set at α = 0.05.

Image-quality metrics were analyzed separately because their availability depended on successful YOLOv8s detection. Observations with RDC = 0 were therefore treated as unavailable (NaN) for bounding-box-dependent image-quality measures rather than assigned artificial zero values, resulting in potentially different sample sizes across conditions. Because these valid detected-region observations represented conditionally detected subsets and were not fully paired across all 55 traffic-sign instances, nonparametric independent-samples comparisons were used. The overall differences in Sharpness were assessed using the Kruskal–Wallis test, followed by pairwise Mann–Whitney U tests with Holm correction.

Accordingly, the statistical analysis distinguishes detector response (RDC) from detection-dependent image-quality characteristics, while accounting for the repeated-measures structure of RDC, avoiding pseudo-replication from consecutive video frames, and appropriately handling unavailable image-quality measurements. Differences in independently recorded real-world rain conditions were interpreted as differences in the observed experimental conditions rather than as effects attributable exclusively to rainfall, because uncontrolled differences in recording sessions may persist.

### 3.9. Reproducibility

Reproducibility was considered throughout the proposed controlled-comparative methodology, with the complete workflow described in Section 3.2, Section 3.3, Section 3.4, Section 3.5, Section 3.6, Section 3.7 and Section 3.8, including model application, weather simulation, frame-level inference, representative-observation selection, image-quality measurement, and statistical analysis.

The YOLOv8s model was previously trained on the publicly available ZND dataset described in Section 3.3 and [16]. No additional training, retraining, fine-tuning, or model adaptation was performed; the same fixed model was applied across all four environmental conditions.

The evaluation used 55 predefined physical traffic-sign instances (S1–S55), consistently associated across conditions using their spatial location, order of appearance, visual characteristics, and sign type. No new manual bounding-box annotations were created, and the evaluation route and physical sign instances were distinct from those represented in the ZND training data, reducing the potential for data leakage.

Frame-level detections were processed using Python, OpenCV, NumPy, and the Ultralytics YOLO implementation. For each sign-condition combination, the highest-confidence valid detection was retained as the representative observation and recorded as RDC. Undetected predefined signs were assigned RDC = 0, while image-quality measurements requiring a detection bounding box were treated as unavailable.

Simulated rain and snow were generated from the clear-weather reference recording using the final parameters in Table 1 and the procedures described in Section 3.4. The same inference and representative-observation procedures were applied consistently across all conditions. The final dataset contained at most 220 sign-condition observations (55 × 4), with all inferential analyses conducted at the traffic-sign level rather than the frame level.

The model-development environment comprised Python 3.10.14, PyTorch 2.4.0, Ultralytics 8.3.36, and CUDA-enabled execution on an NVIDIA Tesla P100 GPU. The final evaluation videos were processed on a local HP Spectre x360 computer (HP Inc., Palo Alto, CA, USA) using the same Ultralytics version (8.3.36) and the previously trained YOLOv8s weights. The final video-processing code did not specify study-specific values for the confidence threshold, non-maximum suppression (NMS) IoU threshold, inference image size, or computational device; therefore, no additional inference-parameter overrides were introduced in the present evaluation. The ZND dataset and model development information are available as described in Section 3.3 and [16]. The evaluation videos and simulated weather sequences are not currently publicly available; however, the principal simulation parameters, processing procedures, evaluation rules, software environment, and statistical methodology are provided to facilitate independent reproduction.

**Figure 3:**
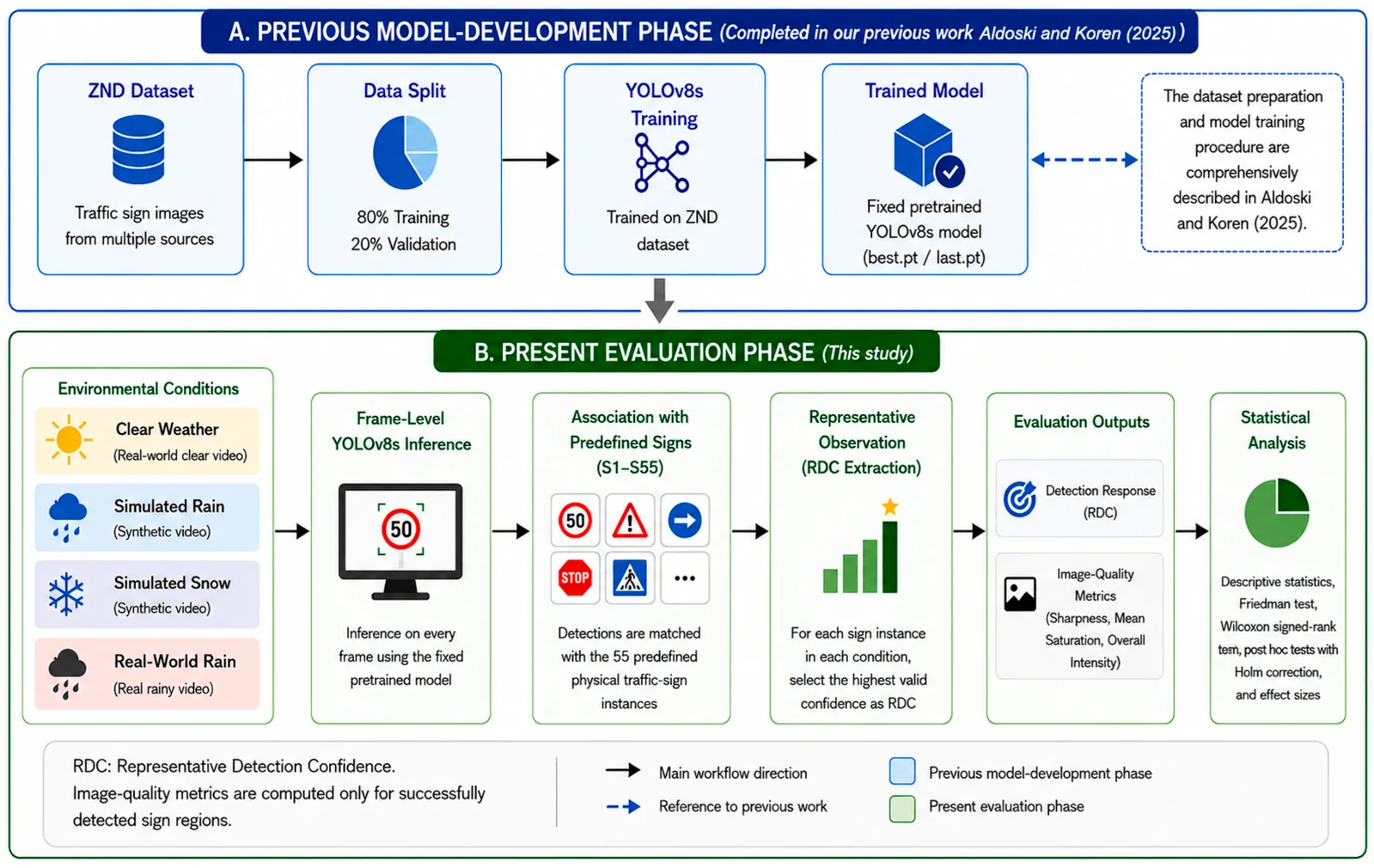
Overall workflow of the study. (**A**) Previous model-development phase based on the ZND dataset and YOLOv8s training. (**B**) Present evaluation phase using the fixed pretrained YOLOv8s model across clear, simulated rain, simulated snow, and real-world rain conditions, followed by frame-level inference, association with the 55 predefined physical traffic-sign instances (S1–S55), RDC extraction, image-quality assessment, and statistical analysis [16]. The five-pointed star indicates the highest valid confidence value selected as the RDC.

Figure 3 summarizes the workflow, distinguishing the previously completed YOLOv8s training phase using ZND from the present testing phase across clear, simulated rain, real-world rain, and simulated snow conditions.

## 4. Results and Discussion

This section presents the results of the controlled-comparative evaluation of the YOLOv8s traffic-sign detector under four environmental conditions: clear, simulated rain, real-world rain, and simulated snow. The analysis is based on 55 predefined physical traffic-sign instances (S1–S55), using the same fixed model and inference procedure across all conditions.

For the simulated conditions, weather transformations were applied to the same clear-weather video sequences, providing a controlled basis for comparison while preserving the underlying scene content. The real-world rain condition was independently recorded on the same road segment using the same vehicle, camera configuration, and traffic-sign inventory; therefore, differences between recording sessions may introduce uncontrolled variability. Accordingly, simulated-condition comparisons provide greater experimental control, whereas comparisons involving real-world rain are interpreted with appropriate caution.

The results are organized around overall detection performance, representative detection confidence (RDC), simulated versus real-world rain, image-quality characteristics, statistical differences across conditions, and exploratory traffic-sign-level patterns.

### 4.1. Overall Detection Performance

Using the proposed controlled-comparative methodology, YOLOv8s performance was evaluated under clear, simulated rain, real-world rain, and simulated snow conditions using the predefined 55 physical traffic-sign instances (S1–S55). RDC was calculated for all 55 instances in each condition, including complete detection failures, whereas image-quality metrics were calculated only for successfully detected sign regions. The resulting summary statistics are presented in Table 2.

Clear conditions produced the highest central RDC values and complete detection coverage, with a mean RDC of 0.825 and all 55 predefined traffic-sign instances detected. Simulated rain produced the lowest mean RDC (0.395) and the highest number of complete detection failures: 30 of 55 instances failed to detect. Real-world rain showed a similar mean RDC (0.408), with 33 of 55 instances detected, whereas simulated snow produced intermediate-to-high performance, with a mean RDC of 0.647 and 45 of 55 instances detected. The broader interquartile ranges and zero-RDC observations under adverse conditions indicate greater heterogeneity in detector response across individual traffic-sign instances.

Image-quality characteristics also varied substantially among the environmental conditions, particularly for Sharpness and Mean Saturation. Clear images exhibited the highest sharpness, while both simulated rain and simulated snow showed markedly lower sharpness. Real-world rain differed from simulated rain in both sharpness and saturation, despite producing a similar aggregate RDC response. Overall Intensity showed comparatively smaller variation between the two rain conditions, but was higher under simulated snow. Because these image-quality measurements were calculated only for successfully detected sign regions, they should be interpreted as conditional characteristics of detected regions rather than as overall image-quality measures for all 55 predefined instances.

Overall, the results indicate that adverse environmental conditions were associated with reduced, more variable detector responses relative to clear conditions, while the magnitude and visual characteristics of degradation varied across conditions. The statistical significance of the differences in RDC is examined in Section 4.5.

### 4.2. Distribution of Representative Detection Confidence

**Figure 4:**
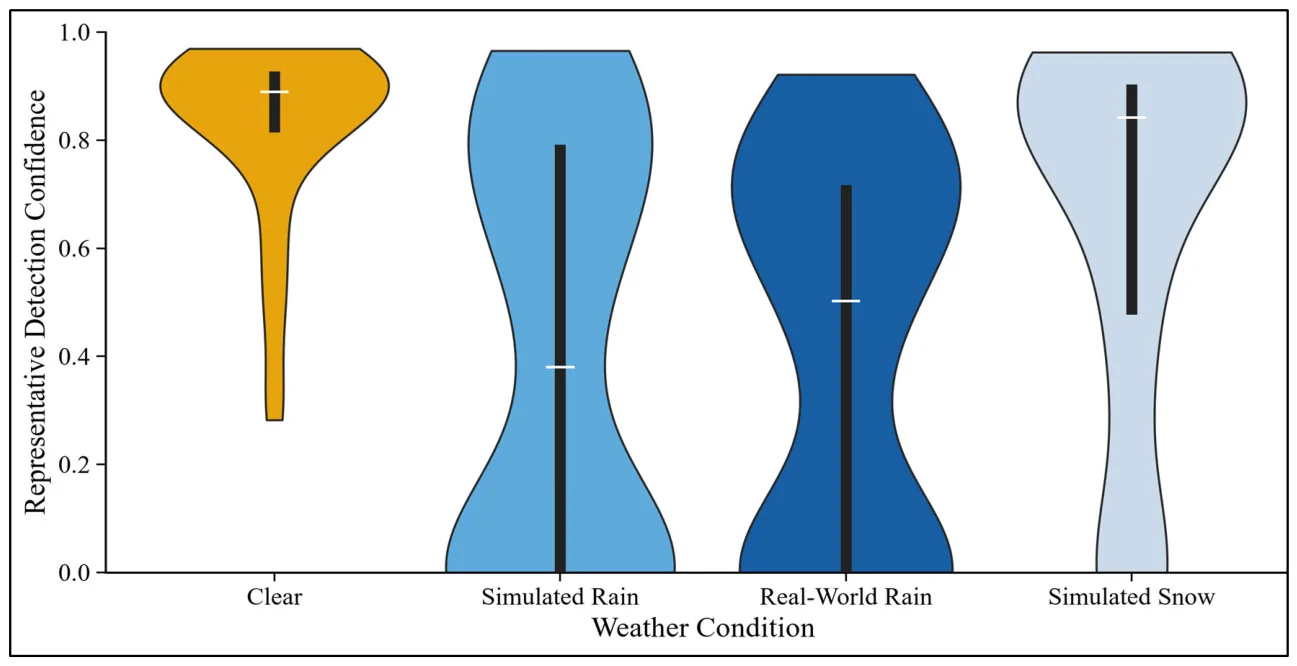
Violin plot of representative detection confidence across the four environmental conditions.

**Figure 5:**
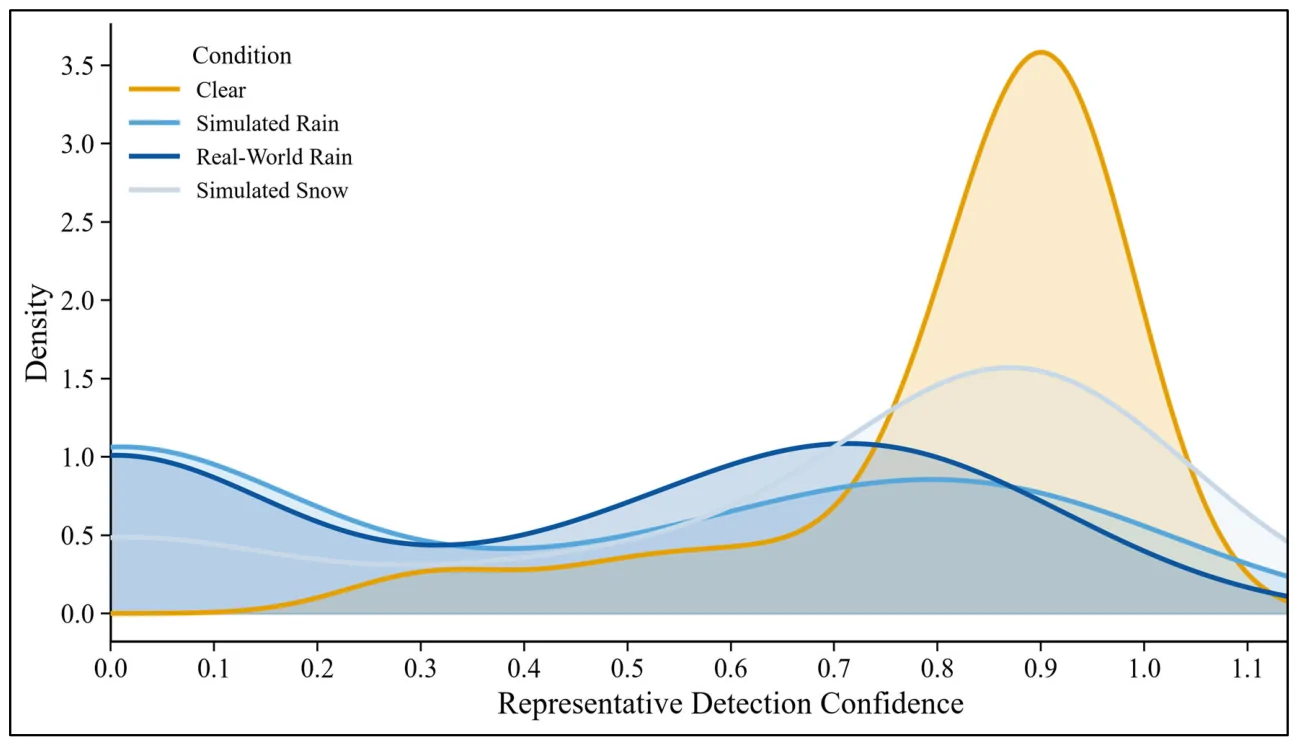
Kernel density estimation of representative detection confidence across the four environmental conditions.

The distribution of RDC values across the 55 predefined traffic-sign instances is shown using violin plots (Figure 4) and kernel density estimation (KDE) (Figure 5). These distributions provide additional information on the central tendency and variability of detection confidence across the four environmental conditions.

Under clear conditions, RDC values were concentrated approximately between 0.8 and 1.0, with a median of 0.889 and an SD of 0.169. All 55 predefined signs had non-zero RDC values. In contrast, simulated rain produced a mean RDC of 0.395 (SD = 0.392) and a median of 0.380, while real-world rain produced a mean RDC of 0.408 (SD = 0.357) and a median of 0.502. Both rainfall conditions showed broader distributions and greater variability than the clear condition.

Simulated snow showed an intermediate pattern, with a mean RDC of 0.647 ± 0.350 and a median of 0.842. Although 10 signs had an RDC of zero under simulated snow, the remaining detected signs were generally concentrated at higher confidence levels. The zero RDC values under the adverse conditions represent complete detector failures and were retained in the analysis.

The inferential analysis identified a statistically significant difference in RDC across the four conditions (Friedman χ^2^(3) = 98.767, *p* < 0.001; Kendall’s W = 0.599). Pairwise Wilcoxon signed-rank comparisons showed significant differences between the clear condition and each adverse-weather condition, and between simulated snow and both rainfall conditions (Holm-adjusted *p* < 0.001). The difference between simulated rain and real-world rain was not significant (Holm-adjusted *p* = 0.770).

Overall, the distributions show that adverse weather increased the variability of detection confidence and introduced complete detection failures, with rainfall producing the broadest reduction in RDC relative to the clear baseline. The statistically comparable RDC responses under simulated and real-world rain provide the basis for the more detailed comparison of their image-quality characteristics in Section 4.3.

### 4.3. Comparative Analysis: Simulated vs. Real-World Rain

The simulated rain and real-world rain conditions were compared to examine whether the controlled simulation produced detector responses comparable to those observed under natural rainfall. Simulated rain was generated from the clear-weather recordings. In contrast, real-world rain was independently recorded on the same road segment using the same camera configuration and predefined traffic-sign inventory. Consequently, the simulated condition provides a more controlled comparison, whereas the real-world condition includes potential variability in recording sessions.

**Figure 6:**
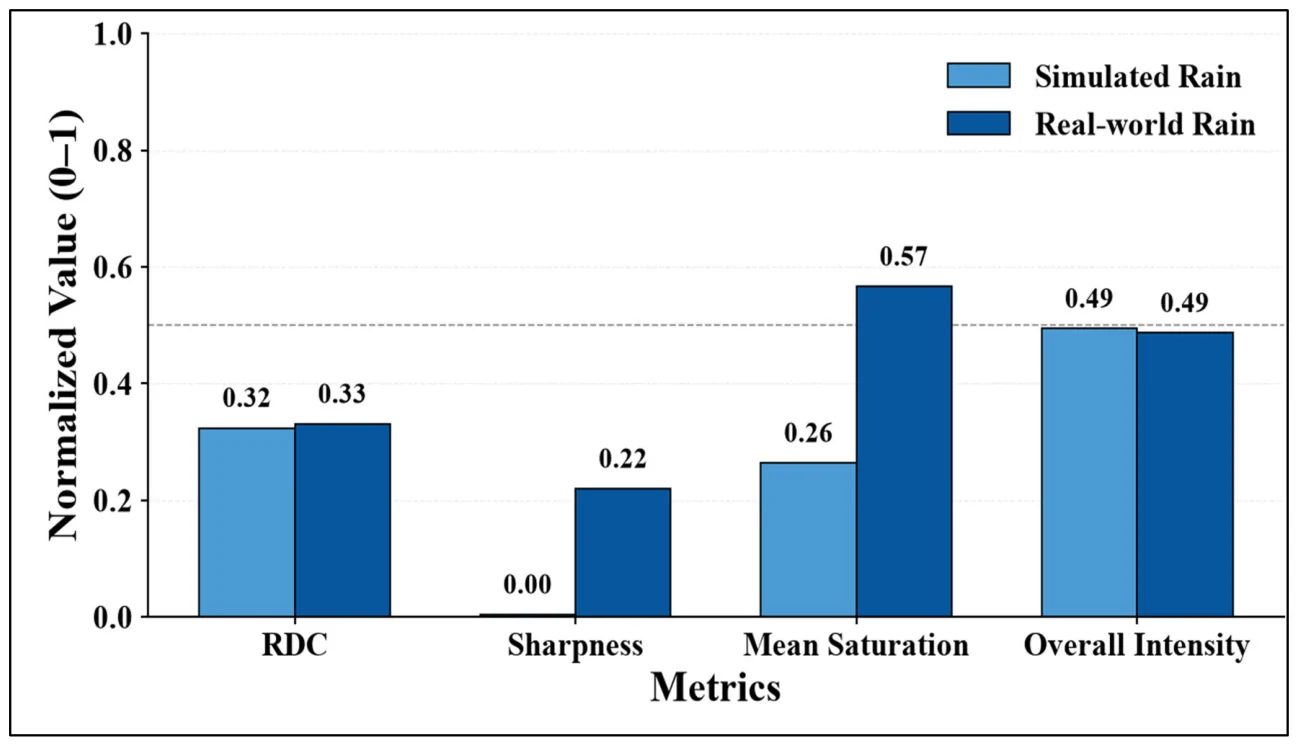
Bounded clear-condition-referenced normalized comparison of representative detection confidence and image-quality metrics between simulated and real-world rain conditions. The dashed horizontal line indicates the 0.50 midpoint of the normalized scale.

Figure 6 presents a normalized comparison of RDC and image-quality metrics between the two rainfall conditions. Normalization was applied only for visual comparison because the evaluated metrics have substantially different numerical scales. A bounded, clear-condition-referenced normalization was used, defined as $N=x/\left(x+x_{clear}\right)$, where x is the mean metric value under the evaluated rainfall condition and $x_{clear}$ is the corresponding mean value under clear conditions. Under this transformation, N=0.5 represents equality with the clear-condition reference, values below 0.5 indicate lower values, and values above 0.5 indicate higher values. The original measurements, variability, and sample sizes are reported in Table 2.

Both rainfall conditions produced substantially lower RDC than the clear condition. However, their direct paired comparison showed no statistically significant difference. Mean RDC was 0.395 ± 0.392 for simulated rain and 0.408 ± 0.357 for real-world rain, with a Holm-adjusted Wilcoxon *p*-value of 0.770. Thus, within the present dataset, simulated and real-world rain produced statistically comparable detector-confidence responses.

**Figure 7:**
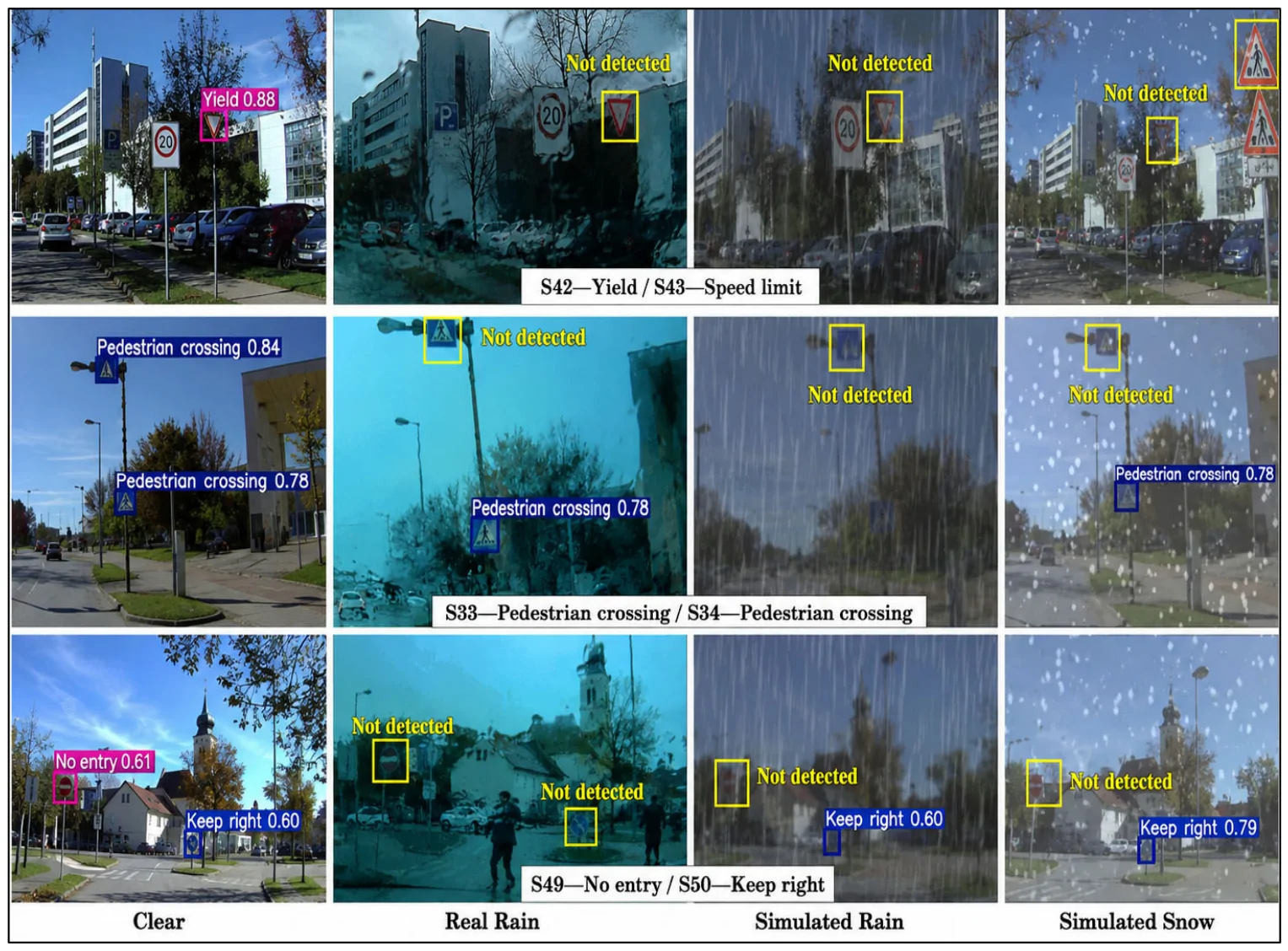
Representative YOLOv8s traffic-sign detection results under clear, simulated rain, real-world rain, and simulated snow conditions. The displayed examples were manually selected to illustrate representative differences in detector response and environmental degradation. The predefined traffic-sign instances are identified by their S-identifiers (S42–S43, S33–S34, and S49–S50) to provide traceability to the S1–S55 evaluation inventory. Yellow boxes indicate the predefined physical sign locations used for traffic-sign instance association and do not represent conventional frame-level ground-truth bounding-box annotations.

Representative detection results are shown in Figure 7. The displayed examples were manually selected to illustrate representative differences in detector response and environmental degradation across the evaluated conditions. The predefined traffic-sign instances are identified by their S-identifiers to provide traceability to the S1–S55 evaluation inventory. Simulated rain produced relatively uniform degradation, including synthetically introduced rain streaks, reduced local contrast, and blur. Real-world rain exhibited greater spatial variability, including localized visual distortions, illumination changes, wet-surface reflections, and varying visibility. Detector failures were observed under both conditions; 30 of the 55 predefined signs had non-zero RDC under simulated rain, compared with 33 under real-world rain. These examples, therefore, illustrate differences in visual degradation rather than evidence that one rainfall condition consistently produced more failures.

The image-quality measurements further distinguish the two rainfall conditions. Mean Sharpness was 34.667 ± 19.933 for simulated rain (*n* = 30) and 2305.288 ± 2698.215 for real-world rain (*n* = 33), with a Mann–Whitney U test showing a statistically significant difference after Holm correction (*p* < 0.001). Because these measurements were available only for successfully detected sign regions, this represents a detected-region comparison rather than a complete paired comparison across all 55 signs. The substantially lower Sharpness under simulated rain is consistent with the applied blur-oriented transformation. In contrast, the higher values under real-world rain indicate that greater local edge information remained within successfully detected regions.

Mean Saturation also differed substantially, with values of 43.817 ± 12.054 for simulated rain and 159.516 ± 79.500 for real-world rain. This difference indicates that the two rainfall conditions produced markedly different saturation characteristics, with real-world rain showing both a higher mean and greater variability. In contrast, Overall Intensity was comparatively similar between simulated rain (113.674 ± 11.186) and real-world rain (110.389 ± 22.805).

The SSIM value of approximately 0.41 for the simulated weather sequences further indicates substantial perceptual change relative to the clear condition. However, it does not provide a direct measure of physical realism or equivalence to real-world rain, as an equivalent SSIM comparison with natural-rain images was not established.

Overall, simulated and real-world rain produced statistically comparable RDC responses despite substantial differences in the image characteristics of successfully detected sign regions. The normalized visualization in Figure 6 further demonstrates that the two rainfall conditions had similar relative RDC values (0.32 for simulated rain and 0.33 for real-world rain). In contrast, their normalized image-quality characteristics differed more substantially, particularly for Sharpness and Mean Saturation. These normalized values should be interpreted as relative visualization measures rather than conventional performance scores or percentages. This indicates that comparable detector-level performance does not necessarily imply comparable image-level appearance. Therefore, simulated rain provides a controlled approximation for detector evaluation, but should not be considered a complete visual substitute for natural rainfall.

### 4.4. Influence of Image Quality on Detection Performance

The results indicate that image-quality characteristics do not exhibit a simple one-to-one relationship with traffic-sign detection performance. The clearest example is Sharpness: although simulated rain produced substantially lower Sharpness than real-world rain (34.667 ± 19.933 vs. 2305.288 ± 2698.215; *p* < 0.001), the corresponding RDC values were not significantly different (0.395 ± 0.392 vs. 0.408 ± 0.357; *p* = 0.770). Thus, a substantial difference in Sharpness among successfully detected sign regions was not accompanied by a significant difference in aggregate detector response.

This result suggests that Sharpness alone is insufficient to characterize detector performance in the present dataset. Moreover, Sharpness, Mean Saturation, and Overall Intensity were calculated only for successfully detected regions, whereas RDC included all 55 predefined traffic-sign instances, including complete detection failures. The two groups of measures, therefore, represent related but distinct aspects of detector behavior.

The qualitative results further indicate that the spatial and visual characteristics of environmental degradation may be important. Simulated rain produced relatively uniform effects such as rain streaks, reduced local contrast, and blur, whereas real-world rain produced more heterogeneous effects, including localized distortions, illumination changes, wet-surface reflections, and spatially varying visibility. A single global image-quality measure does not fully represent these differences.

Mean Saturation also differed substantially between simulated and real-world rain (43.817 ± 12.054 vs. 159.516 ± 79.500), whereas Overall Intensity was comparatively similar (113.674 ± 11.186 vs. 110.389 ± 22.805). However, because no direct statistical relationship between these image-quality measures and RDC was established, these findings should be interpreted as differences in image characteristics rather than causal effects on detection performance.

Overall, the results show that detector-level performance and image-level degradation should not be treated as interchangeable measures. Similar RDC responses can occur despite substantial differences in Sharpness and Mean Saturation, indicating that no single image-quality metric provides a sufficient surrogate for traffic-sign detection robustness.

### 4.5. Statistical Analysis of Detection Performance

The inferential analysis used one RDC value for each of the 55 predefined traffic-sign instances under each environmental condition, resulting in 55 matched sign instances across four conditions. Matching was based on physical traffic-sign identity (S1–S55), rather than frame-to-frame correspondence, thereby avoiding the treatment of temporally correlated video frames as independent observations.

Because the same 55 traffic-sign instances were evaluated across all four conditions and non-normal empirical distributions bound RDC, the Friedman test was used for the overall comparison, followed by paired Wilcoxon signed-rank tests for post hoc comparisons. Holm correction was applied to the six pairwise comparisons. The results are presented in Table 3 and Table 4.

The Friedman test identified a statistically significant difference in RDC across the four environmental conditions (χ^2^(3) = 98.767, *p* < 0.001), with Kendall’s W = 0.599. Pairwise comparisons showed that clear conditions differed significantly from simulated rain, real-world rain, and simulated snow, while simulated snow also differed significantly from both rainfall conditions (Holm-adjusted *p* < 0.001).

In contrast, simulated rain and real-world rain did not differ significantly in RDC (Holm-adjusted *p* = 0.770). This result supports the statistical comparability of their detector-level responses within the present dataset. Still, it does not establish statistical equivalence, physical realism, or identical visual degradation between the two rainfall conditions.

The corrected mean RDC values give the descriptive ordering clear (0.825) > simulated snow (0.647) > real-world rain (0.408) > simulated rain (0.395). However, this ordering should be interpreted together with the inferential results; in particular, the small difference between the two rainfall means was not statistically significant.

Overall, the inferential analysis confirms a significant effect of environmental conditions on RDC and demonstrates that the two rainfall conditions produced statistically comparable detector responses. The results, therefore, support distinguishing statistically significant condition differences from descriptive differences in mean RDC.

### 4.6. Impact of Traffic Sign Characteristics

**Figure 8:**
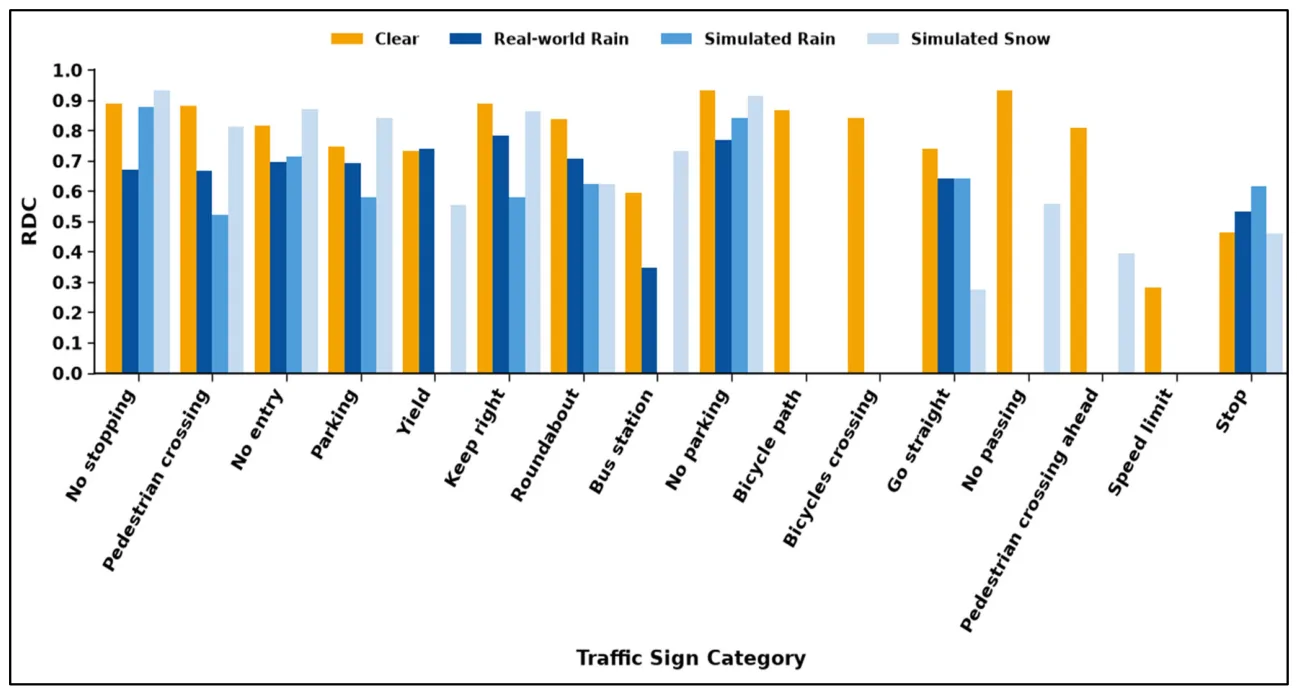
RDC across traffic sign categories under clear, simulated rain, real-world rain, and simulated snow conditions.

To examine traffic-sign-level variation in detector response, RDC was analyzed across individual traffic-sign categories, as shown in Figure 8. Because the 55 predefined traffic-sign instances were unevenly distributed among categories, these results are presented as descriptive and exploratory observations.

Figure 8 presents the average RDC for each traffic-sign category under clear, real-world rain, simulated rain, and simulated snow conditions.

Under clear conditions, relatively high RDC values were observed for No stopping, keep right, no parking, Bicycle path, and No passing. In contrast, the bus station and Speed limit showed comparatively lower values. Under adverse conditions, the magnitude of degradation varied among categories. For example, no stopping maintained relatively high detection under simulated rain and simulated snow, whereas pedestrian crossing showed a more pronounced reduction under simulated rain. The roundabout and Bus station also exhibited different responses across conditions. These patterns indicate heterogeneous responses among the evaluated traffic-sign instances, but the uneven category sizes do not support general category-wide conclusions.

Several category–condition combinations have no displayed bar. These represent unavailable valid observations rather than RDC = 0 and should be distinguished from genuine zero-RDC detector failures. The comparison between simulated and real-world rain also showed no consistent category-level pattern. Some categories had lower RDC under simulated rain, whereas others showed the opposite or insufficient observations for comparison.

**Figure 9:**
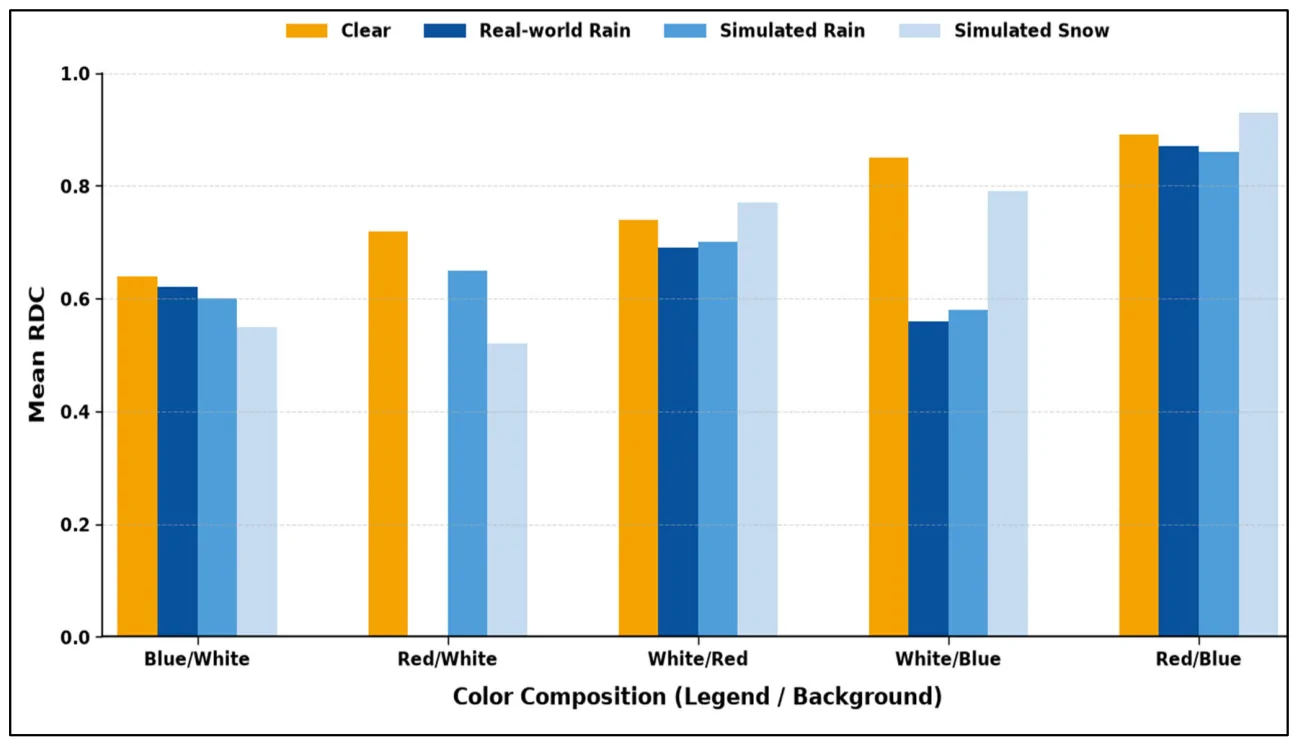
RDC by traffic sign color composition under different environmental conditions.

To further examine visual appearance, the RDC was analyzed in terms of traffic-sign color composition (Figure 9).

The Red/Blue and White/Blue groups showed relatively high RDC under clear conditions, while Blue/White showed a comparatively lower value. Red/Blue remained relatively high across the evaluated environmental conditions, whereas White/Blue showed a more pronounced reduction under rainfall, particularly real-world rain. Blue/White showed a more moderate decline. White/Red also showed relatively high detection performance across the available conditions, while fewer condition-specific observations represented Red/White.

The color-composition results should be interpreted cautiously because group sizes were limited and uneven. Although stronger color contrast may contribute to discriminative information, the observed RDC may also reflect sign shape, graphical content, size, background, illumination, and spatial context. Thus, these results indicate exploratory associations rather than independent effects of color composition. As in Figure 8, an absent bar indicates an unavailable observation and not an RDC of zero.

No separate inferential tests were performed for traffic-sign categories or color-composition groups because of the small and uneven numbers of predefined physical sign instances. Accordingly, Figure 8 and Figure 9 provide exploratory evidence of traffic-sign-level heterogeneity rather than statistically established category- or color-specific effects.

Overall, the results show that adverse-weather effects were not uniform across the evaluated traffic signs. Differences among categories and color compositions indicate that aggregate RDC can conceal variation at the individual-sign level; however, the present 55-sign inventory is insufficient to establish independent causal effects of sign category or color composition.

### 4.7. Implications for Autonomous Driving Systems

The findings have important implications for the robustness of vision-based traffic-sign detection in autonomous driving applications. The statistically significant differences in RDC across environmental conditions demonstrate that adverse weather can substantially influence detector response and its variability across traffic-sign instances. However, the absence of a significant RDC difference between simulated and real-world rain indicates that neither rainfall is statistically superior or inferior in the present dataset.

An important implication is that comparable detector-level performance does not necessarily indicate comparable image-level degradation. Although simulated and real-world rain produced statistically comparable RDC responses, their image characteristics and visual effects differed substantially. This indicates that synthetic weather can provide a controlled approximation of adverse-weather effects for detector evaluation. At the same time, real-world data remain important for capturing the heterogeneous visual characteristics of natural environmental conditions.

The object-level results further indicate that aggregate performance measures may conceal vulnerabilities affecting individual traffic-sign instances. Differences among evaluated sign categories and color compositions suggest that perception robustness can vary with the visual and structural characteristics of individual signs. Therefore, combining aggregate evaluation with object-level analysis can provide a more informative assessment of potential perception weaknesses.

From an autonomous-driving perspective, these findings support the use of both controlled synthetic weather data and representative real-world adverse weather data in perception-system development and validation. Future work should investigate larger, more diverse datasets; systematic validation across synthetic and natural weather conditions; weather-diverse training and augmentation strategies; and complementary sensing modalities such as LiDAR and radar.

Overall, simulated weather should be regarded as a controllable and reproducible benchmarking tool rather than a complete replacement for natural-weather evaluation. The combined use of controlled simulation, real-world weather data, image-quality assessment, and object-level analysis provides a stronger basis for evaluating environmental robustness in vision-based traffic-sign detection systems.

### 4.8. Summary of Key Findings

The controlled-comparative evaluation demonstrated that environmental conditions substantially influence YOLOv8s traffic-sign detection performance. In contrast, the use of the same predefined traffic-sign instances and controlled scene characteristics provides a consistent basis for comparison. Clear conditions produced the highest mean RDC (0.825), followed by simulated snow (0.647), real-world rain (0.408), and simulated rain (0.395). The Friedman test confirmed a statistically significant overall difference among the four conditions (χ^2^(3) = 98.767, *p* < 0.001, Kendall’s W = 0.599). However, simulated and real-world rain did not differ significantly in RDC (Holm-adjusted *p* = 0.770), indicating statistically comparable aggregate detector responses rather than a performance hierarchy between the two rainfall conditions.

The analyses also demonstrated that detector-level performance and image-level degradation should not be treated as interchangeable. Simulated and real-world rain produced substantially different image-quality characteristics and visual degradation patterns despite their statistically comparable RDC responses. In addition, the broader RDC distributions and object-level variation observed under adverse conditions indicate that aggregate performance can conceal heterogeneous responses among individual traffic-sign instances.

Taken together, the findings support the use of simulated weather as a controlled and reproducible tool for adverse-weather benchmarking, while demonstrating the continued importance of real-world weather data for validation. The proposed methodology therefore provides a framework for evaluating environmental robustness at both the aggregate detector level and the traffic-sign-instance level, with direct relevance to the development and evaluation of reliable vision-based traffic-sign detection systems for autonomous-driving applications.

## 5. Conclusions

This study evaluated the environmental robustness of a previously trained YOLOv8s traffic-sign detector under four conditions: clear weather, simulated rain, real-world rain, and simulated snow. The evaluation used a predefined inventory of 55 physical traffic-sign instances and a controlled-comparative methodology designed to minimize scene-level variability in the simulated-weather comparisons. The real-world rain condition was independently recorded on the same road segment using the same vehicle and camera configuration and the same predefined sign inventory, while retaining the additional variability associated with a separate recording session.

The results demonstrated a statistically significant difference in RDC among the four environmental conditions (Friedman χ^2^(3) = 98.767, *p* < 0.001; Kendall’s W = 0.599). Clear conditions produced the highest mean RDC (0.825), followed by simulated snow (0.647), real-world rain (0.408), and simulated rain (0.395). However, simulated and real-world rain did not differ significantly in RDC (Holm-adjusted *p* = 0.770). Thus, the observed mean ordering should not be interpreted as evidence of a statistically superior rainfall condition.

The comparison between simulated and real-world rain further demonstrated that statistically comparable detector-level responses can occur despite substantially different image-level characteristics. In particular, the rainfall conditions differed markedly in Sharpness and Mean Saturation, whereas Overall Intensity was comparatively similar. Because these image-quality measurements were calculated only for successfully detected sign regions, they characterize the image characteristics of detected regions rather than overall image quality across all predefined traffic-sign instances.

The distributional and object-level analyses additionally showed that adverse-weather effects were heterogeneous across the evaluated traffic-sign instances. Greater variation in RDC and genuine zero-RDC observations under adverse conditions indicates that aggregate performance measures alone may conceal vulnerabilities affecting individual signs. The observed differences among traffic-sign categories and color compositions should therefore be regarded as exploratory evidence of heterogeneous detector responses rather than independent causal effects of sign shape or color.

The principal contribution of the study is therefore the controlled-comparative evaluation methodology rather than a modification of the YOLOv8s architecture. By combining controlled synthetic-weather conditions with an independently recorded natural-rain condition and evaluating a common predefined traffic-sign inventory, the methodology provides a structured basis for distinguishing detector-level response from image-level environmental variation. The findings demonstrate that simulated weather can provide a useful and reproducible approximation for adverse-weather benchmarking, but cannot fully replace real-world environmental evaluation.

Several limitations should be considered when interpreting these findings. The RDC measure represents the highest valid detector confidence observed for each predefined sign-condition combination. It therefore does not establish average confidence, worst-case confidence, detection persistence, or frame-level temporal consistency. In addition, image-quality comparisons were conditional on successful detections and were not fully paired across conditions. The study also used a fixed pretrained YOLOv8s detector and a geographically limited traffic-sign inventory, which restricts generalization beyond the present experimental setting.

Future research should extend the methodology by using larger, more diverse real-world datasets, additional weather conditions and locations, complete frame-level ground truth, and multiple object-detection architectures. Such extensions could also enable temporal consistency and detection-persistence analyses, and support the evaluation of multimodal perception systems that incorporate complementary sensors such as LiDAR and radar.

Overall, the study demonstrates that controlled synthetic-weather evaluation can provide valuable, reproducible evidence of weather-related changes in traffic-sign detector response. At the same time, real-world adverse-weather data remains essential for assessing environmental variability that may not be reproduced by simulation. The proposed methodology provides a practical foundation for a more systematic and generalizable evaluation of the robustness of vision-based traffic-sign detection in autonomous driving applications.

### 5.1. Limitations

Although the proposed framework provides a controlled comparison of traffic-sign detection under different environmental conditions, several limitations should be considered.

First, the simulated weather conditions reproduce selected visual effects through controlled image transformations rather than complete physical weather processes. Natural rain and snow involve additional effects such as stochastic precipitation, illumination variation, atmospheric scattering, reflections, lens contamination, accumulation, and deposition. Therefore, the simulated conditions should be regarded as controlled approximations rather than complete physical representations of adverse weather.

Second, the evaluation was limited to clear weather, simulated rain, real-world rain, and simulated snow using RGB imagery. Other challenging scenarios, including dense fog, heavy snowfall, nighttime precipitation, and mixed weather, were not investigated. Although the same road segment, camera configuration, and predefined S1–S55 traffic-sign inventory were maintained, the real-world rain recording was acquired independently and therefore included uncontrolled session-related variability. The observed reversal in Mean Saturation further highlights a limitation of the simulated-rain transformation: Mean Saturation decreased from 122.128 under clear conditions to 43.817 under simulated rain, whereas real-world rain produced a higher mean value of 159.516. This difference indicates that the simulation does not reproduce the direction or magnitude of all photometric changes associated with natural rainfall.

Third, an inferential analysis was performed at the predefined traffic-sign-instance level, with a single RDC value retained for each sign-condition combination. RDC represents the maximum valid confidence observed during the available observation of a sign and therefore reflects the strongest observed detector response rather than average or worst-case confidence. Because the frame-level records do not independently establish the complete physical visibility interval of each sign, conventional detection-persistence or detection-frequency measures cannot be calculated reliably. Consequently, RDC does not characterize the temporal persistence of detection: a sign detected with high confidence in only one frame could receive an RDC comparable to that of a sign detected consistently throughout its observable period. A valid persistence or detection-frequency rate would require independent frame-level identification of the first and last physically visible frames for each sign. Accordingly, the present analysis does not establish frame-to-frame temporal consistency. Future evaluations should therefore establish the complete physical visibility interval for each traffic-sign instance using independent frame-level ground truth and complement RDC with detection frequency, consecutive-detection duration, and temporal-consistency measures.

Fourth, image-quality metrics were available only for successfully detected traffic-sign regions, resulting in unequal sample sizes across conditions. These measurements, therefore, represent characteristics of detected regions rather than of the complete image set, and the observed differences should not be interpreted as direct causal effects of image quality on detection performance.

Fifth, conventional object-detection metrics, including mAP, precision, recall, and F1-score, were not calculated because rigorous evaluation would require complete frame-level ground-truth bounding boxes and explicit detection-to-ground-truth matching. The present study instead used the predefined inventory of 55 physical traffic-sign instances and RDC for controlled environmental comparison. It should therefore not be interpreted as a conventional object-detection benchmark. In addition, although the evaluated physical signs differed from those used to develop the pretrained YOLOv8s model, the ZND dataset and evaluation protocol originate from the same broader geographic area; therefore, complete geographic independence cannot be assumed.

Finally, the study used a fixed pretrained YOLOv8s detector without introducing any new trainable components; therefore, architecture-level ablation was outside its scope. Although the proposed evaluation methodology can, in principle, be applied to other detectors and multimodal systems, their responses to adverse weather may differ. The findings should consequently not be generalized directly beyond YOLOv8s without additional validation.

### 5.2. Future Research Directions

The proposed controlled-comparative methodology provides several directions for future research.

Future studies should incorporate larger, more diverse real-world adverse-weather datasets that cover conditions such as dense fog, heavy snowfall, nighttime precipitation, and mixed weather. Repeated recordings across different locations, traffic conditions, illumination levels, precipitation intensities, and acquisition sessions would improve assessment of external validity and generalizability.

Synthetic weather generation could be further investigated using physics-based rendering, generative artificial intelligence, and data-driven weather models that represent dynamic precipitation, water accumulation, atmospheric effects, and lens contamination. Such approaches should be evaluated using both visual characteristics and detector-level responses to determine whether synthetic conditions provide meaningful approximations of natural weather.

The framework could also be extended to multimodal perception systems by examining cameras, LiDAR, radar, and sensor fusion in adverse weather conditions. Weather-aware learning, domain adaptation, uncertainty-aware detection, and context-aware approaches could be evaluated using traffic-sign-level RDC and, where appropriate, frame-level detection records. When complete frame-level ground truth is available, future studies should also incorporate false-positive analysis and conventional metrics such as precision, recall, F1 Score, and mAP.

Further research should investigate the relationship between detector response and image quality using measurements obtained independently of successful detection. Predefined regions of interest could enable measurement of image-quality characteristics for both detected and non-detected sign instances, while repeated observations could support statistical approaches that explicitly account for temporal dependence.

Finally, the methodology should be evaluated across different traffic-sign standards, geographic regions, image resolutions, compression levels, and detector architectures. Cross-dataset and cross-detector validation would help determine whether the observed relationships among environmental conditions, RDC distributions, and image characteristics remain consistent beyond the present experimental setting and could support the development of more standardized and generalizable adverse-weather evaluation protocols.

## Figures and Tables

**Table 1:** Principal parameters used in the final synthetic-weather simulation implementation.

Weather Condition	Simulation Component	Principal Settings
**Rain**	Rain streaks	≈1000 streaks/frame; 10–20 pixels length; 2 pixels thickness; random slant −10 to −4 pixels; Gaussian blur 5 × 5
	Lens-water effects	35 droplets/frame; radius 10–30 pixels; opacity 0.10–0.30; Gaussian blur 9 × 9, σ = 5
	Visibility degradation	Fog intensity = 0.40; global Gaussian blur 11 × 11, σ = 3
	Wet road and displacement	15 localized elements; blending weight = 0.10; displacement ±2 pixels
**Snow**	Snow particles	700 particles/frame; radius 2–6 pixels; Gaussian blur 5 × 5
	Lens-frost effects	30 specks/frame; radius 8–25 pixels; opacity 0.10–0.25; Gaussian blur 11 × 11, σ = 3
	Visibility degradation	Fog intensity = 0.40; global Gaussian blur 9 × 9, σ = 2.5
	Image displacement	±2 pixels in horizontal and vertical directions

**Table 2:** Detection performance and image-quality metrics across environmental conditions.

Environmental Condition	RDC (Median [Q1–Q3]; Mean ± SD)	Min–Max	Detected/55 (%)	Sharpness: Laplacian Variance (Mean ± SD)	Mean Saturation: HSV-S (0–255; Mean ± SD)	Overall Intensity: HSV-V (0–255; Mean ± SD)
**Clear**	0.889 [0.815–0.927]; 0.825 ± 0.169	0.282–0.969	55/55 (100.0%)	8179.7 ± 6185.3	122.1 ± 30.6	116.3 ± 40.7
**Simulated Rain**	0.380 [0.000–0.792]; 0.395 ± 0.392	0.000–0.965	30/55 (54.5%)	34.7 ± 19.9	43.8 ± 12.1	113.7 ± 11.2
**Real-World Rain**	0.502 [0.000–0.718]; 0.408 ± 0.357	0.000–0.921	33/55 (60.0%)	2305.3 ± 2698.2	159.5 ± 79.5	110.4 ± 22.8
**Simulated Snow**	0.842 [0.477–0.904]; 0.647 ± 0.350	0.000–0.963	45/55 (81.8%)	51.7 ± 36.0	44.2 ± 13.7	143.1 ± 16.0

Note: RDC, representative detection confidence; Q1 and Q3, first and third quartiles; SD, standard deviation; HSV-S, saturation channel of the 8-bit HSV representation; HSV-V, value channel of the 8-bit HSV representation. Sharpness was quantified as the variance of the Laplacian response and is therefore an image-derived sharpness index rather than a physical unit. Mean Saturation and Overall Intensity are reported on the 0–255 digital channel scale. RDC = 0 indicates that no valid YOLOv8s detection/classification was recorded for the corresponding predefined traffic-sign instance under that environmental condition. Detection coverage represents the number of predefined traffic-sign instances with RDC > 0 out of the total 55 instances. Image-quality measures were calculated only for successfully detected traffic-sign regions; therefore, their sample sizes were 55, 30, 33, and 45 for clear, simulated rain, real-world rain, and simulated snow, respectively.

**Table 3:** Friedman test results for the RDC across environmental conditions.

Test	χ^2^	df	*p*-Value	Kendall’s W	Significance
**Friedman test**	98.767	3	<0.001	0.599	Significant

**Table 4:** Wilcoxon signed-rank post hoc comparisons of RDCs with Holm correction.

Comparison	Wilcoxon W	Raw *p*-Value	Holm-Adjusted *p*-Value	Effect Size	Significant
**Clear vs** **.** **Simulated Rain**	27.000	<0.001	<0.001	0.965	Yes
**Clear vs. Real-World Rain**	37.000	<0.001	<0.001	0.952	Yes
**Clear vs. Simulated Snow**	0.000	<0.001	<0.001	1.000	Yes
**Simulated Rain vs. Real-World Rain**	204.000	0.770	0.770	−0.062	No
**Simulated Rain vs. Simulated Snow**	60.000	<0.001	<0.001	−0.884	Yes
**Real-World Rain vs. Simulated Snow**	97.000	<0.001	<0.001	−0.813	Yes

## Data Availability

The ZND dataset used in our study is freely available online at https://www.kaggle.com/datasets/endaziar/znd-dataset (accessed on 20 April 2026).

## Abbreviations

The following abbreviations are used in this manuscript:

AV	Autonomous Vehicle
ADAS	Advanced Driver Assistance Systems
GTSRB	German Traffic Sign Recognition Benchmark
mAP	Mean Average Precision
NaN	Not a Number
RDC	Representative Detection Confidence
SSIM	Structural Similarity Index Measure
YOLO	You Only Look Once
ZND	Ziyad–N Dataset
